# The Evolution of Bat Vestibular Systems in the Face of Potential Antagonistic Selection Pressures for Flight and Echolocation

**DOI:** 10.1371/journal.pone.0061998

**Published:** 2013-04-24

**Authors:** Kalina T. J. Davies, Paul J. J. Bates, Ibnu Maryanto, James A. Cotton, Stephen J. Rossiter

**Affiliations:** 1 School of Biological and Chemical Sciences, Queen Mary University of London, London, United Kingdom; 2 Department of Palaeontology, Natural History Museum, London, United Kingdom; 3 Harrison Institute, Sevenoaks, Kent, United Kingdom; 4 Indonesian Institute of Sciences (LIPI) and Museum Bogoriense, Cibinong, West Java, Indonesia; Friedrich-Schiller-University Jena, Germany

## Abstract

The vestibular system maintains the body’s sense of balance and, therefore, was probably subject to strong selection during evolutionary transitions in locomotion. Among mammals, bats possess unique traits that place unusual demands on their vestibular systems. First, bats are capable of powered flight, which in birds is associated with enlarged semicircular canals. Second, many bats have enlarged cochleae associated with echolocation, and both cochleae and semicircular canals share a space within the petrosal bone. To determine how bat vestibular systems have evolved in the face of these pressures, we used micro-CT scans to compare canal morphology across species with contrasting flight and echolocation capabilities. We found no increase in canal radius in bats associated with the acquisition of powered flight, but canal radius did correlate with body mass in bat species from the suborder Yangochiroptera, and also in non-echolocating Old World fruit bats from the suborder Yinpterochiroptera. No such trend was seen in members of the Yinpterochiroptera that use laryngeal echolocation, although canal radius was associated with wing-tip roundedness in this group. We also found that the vestibular system scaled with cochlea size, although the relationship differed in species that use constant frequency echolocation. Across all bats, the shape of the anterior and lateral canals was associated with large cochlea size and small body size respectively, suggesting differential spatial constraints on each canal depending on its orientation within the skull. Thus in many echolocating bats, it seems that the combination of small body size and enlarged cochlea together act as a principal force on the vestibular system. The two main groups of echolocating bats displayed different canal morphologies, in terms of size and shape in relation to body mass and cochlear size, thus suggesting independent evolutionary pathways and offering tentative support for multiple acquisitions of echolocation.

## Introduction

During their adaptive radiation to occupy new environments, mammals have faced multiple simultaneous selection pressures that have shaped their ecology, locomotion and sensory modality. Many of these pressures are likely to have been antagonistic, such that changes conferring fitness advantages in one aspect may at the same time be disadvantageous in another. This kind of fitness trade-off between different selective pressures is a central concept in evolutionary biology, but the best-studied examples are of life-history trade-offs (e.g. [1], [2]). In contrast, documented examples of trade-offs in morphological traits are surprisingly rare but could provide insights into how selection can act in the face of constraints (e.g. [3]–[5]).

Here we tested for evidence of potential evolutionary constraints on the vertebrate vestibular system, which incorporates three approximately orthogonally oriented semicircular canals (anterior, posterior and lateral) that are responsible for monitoring angular acceleration of the head, and thus maintaining the body’s sense of balance [6]. The vestibular system also stabilises gaze by means of the vestibulo-ocular and the vestibulo-collic reflexes that, respectively, act on the extraocular and neck musculature to counteract head movements and maintain the angle of vision [6], [7]. In mammals the bony inner ear labyrinth, which consists of the cochlea and the vestibular system, is housed within the petrosal bone in the ventral, posterior part the skull. The cochleae – which in some mammal species may be partly enclosed by auditory bullae – are located more ventrally and rostrally, whereas the vestibular system projects upwards, outwards and backwards into the skull. The relative orientation of the three canals, with respect to each other, as well as to the extraocular muscles and head posture, may all be under functional constraint [6], [8]–[10]. Alignment of the lateral semicircular canal is speculated to be the most tightly linked to head posture, when either at rest or during locomotion (e.g. [11]), with optimum canal plane alignment thought to approximate the horizon, or with a slight incline (reviewed in [6]). The posterior canal plane lies perpendicular to that of the lateral canal, and projects dorso-laterally with respect to the skull. Therefore while the lateral and posterior canals project laterally, the plane of the anterior canal projects upwards towards the top of the skull. Several functional and spatial constraints are thought to act on the vestibular system. For example, evidence from humans suggests that reorientation of the petrosal part of the skull, putatively associated with either brain expansion or a bipedal gait, can influence labyrinth orientation, although the planar orientations of the canals are conserved [9]. In some vertebrates (e.g. pterosaurs and primates [11], [12]) the petrosal lobe of the cerebellar paraflocculus, which is involved in processing eye movements during locomotion, has been shown to affect the size of semicircular canals. As the flocculus sits within the canals, expansion of this brain region can be associated with an increase in semicircular canal size [11], [12].

Previous studies have reported overall negative allometric relationships between semicircular canal size and body mass (e.g. [13]–[15]), as well as more radical morphological changes associated with major transitions in locomotion (e.g. [16], [17]). For example, the canals of cetaceans are massively reduced, perhaps due to their aquatic locomotion coupled with reduced neck mobility [17], [18], whereas subterranean mammals tend to have either substantially larger canals or wider lumens than terrestrial taxa have [19], [20]. A proposed explanation for the latter relationship is that increased size may confer greater sensitivity [21], so meeting the demands of navigation through their subterranean niche without visual cues [20]. More generally, within both birds and mammals, canal size appears to be positively correlated with agility [15], [22], [23] and such relationships have been used to infer the ecology of extinct specimens based on their inner ear morphology (e.g. [22], [24], [25]). However, while some studies have stated a clear link between semicircular canal structure and locomotory style in birds (e.g. [26]) others have suggested the relationship is not so obvious (e.g. [27]). Furthermore, comparing the results of studies of avian canals is somewhat limited by their use of different measurements as well as contrasting methods for controlling for body size. Overall, the links between agility and canal size have been contentious (e.g. [28]) and undoubtedly canal morphology will have been affected by many additional factors such as phylogenetic and mechanical constraints (e.g. [29]–[31]).

Bats are unique among mammals in having evolved powered (and often highly manoeuvrable) flight [32] which places unusual demands on their vestibular systems. Aerobatic manoeuvrability and slow flight correlate positively with the size and assumed mechanical sensitivity of semicircular canals in flighted birds [22], so that we might expect to see similar relationships in bats. Yet unlike birds, bats have also uniquely evolved laryngeal echolocation, and have undergone associated massive expansion of their cochleae for ultrasonic hearing [33]. Since the semicircular canals are physically attached to the cochlea within the limited space of the petrosal bone, the vestibular system of bats might therefore be under antagonistic selection pressures for flight and echolocation performance. Indeed, a constraining effect of skull size on semicircular morphology has been widely speculated [12], [23], [28], [29]. In this context, it is especially intriguing that horseshoe bats (Rhinolophidae), which are characterised by particularly manoeuvrable and slow flight, also possess some of the largest cochleae of all bats, probably associated with the evolution of constant frequency (CF) echolocation in this lineage [33]. If semicircular canal size and/or shape are indeed influenced by cochlea hypertrophy in echolocating bats, then canal morphology could shed light on the evolution of laryngeal echolocation, which occurs in two divergent clades. Current phylogenetic and fossil evidence suggest that powered flight evolved before echolocation in bats, but whether or not echolocation evolved more than once or was lost by non-echolocating Old World fruit bats (Pteropodidae), is unresolved [34]–[37]. Previous studies on bat vestibular systems have considered single species and thus provide few clues into the effects and origins of echolocation [38]–[40].

To determine the evolutionary consequences of echolocation and flight on the morphology of semicircular canals in bats, we conducted high-resolution micro-computed tomography (µCT) scans of inner ear labyrinths of a range of bats and compared our results to data from non-flying mammals. Here we use a comparative approach to examine how the vestibular systems of bats have been influenced by functional constraints associated with flight and echolocation. We first predicted that as the only mammals capable of powered flight all bats would have proportionally larger semicircular canals compared to those of non-flying mammals. Second we predicted that the semicircular canals of echolocating bats would show modifications (either deviations in size or shape) compared to those of non-echolocating bats, due to physical constraints imposed by enlarged cochleae. For example, we might expect echolocating bats to have semicircular canals that show greater deviations from circularity. Finally, we predicted that the relative size of semicircular canal size canal should correlate positively with wing morphology, and thus flight ability, in the face of any detected constraints.

## Materials and Methods

### Study Sample and Acquisition of µCT-scan Data

**Figure 1:**
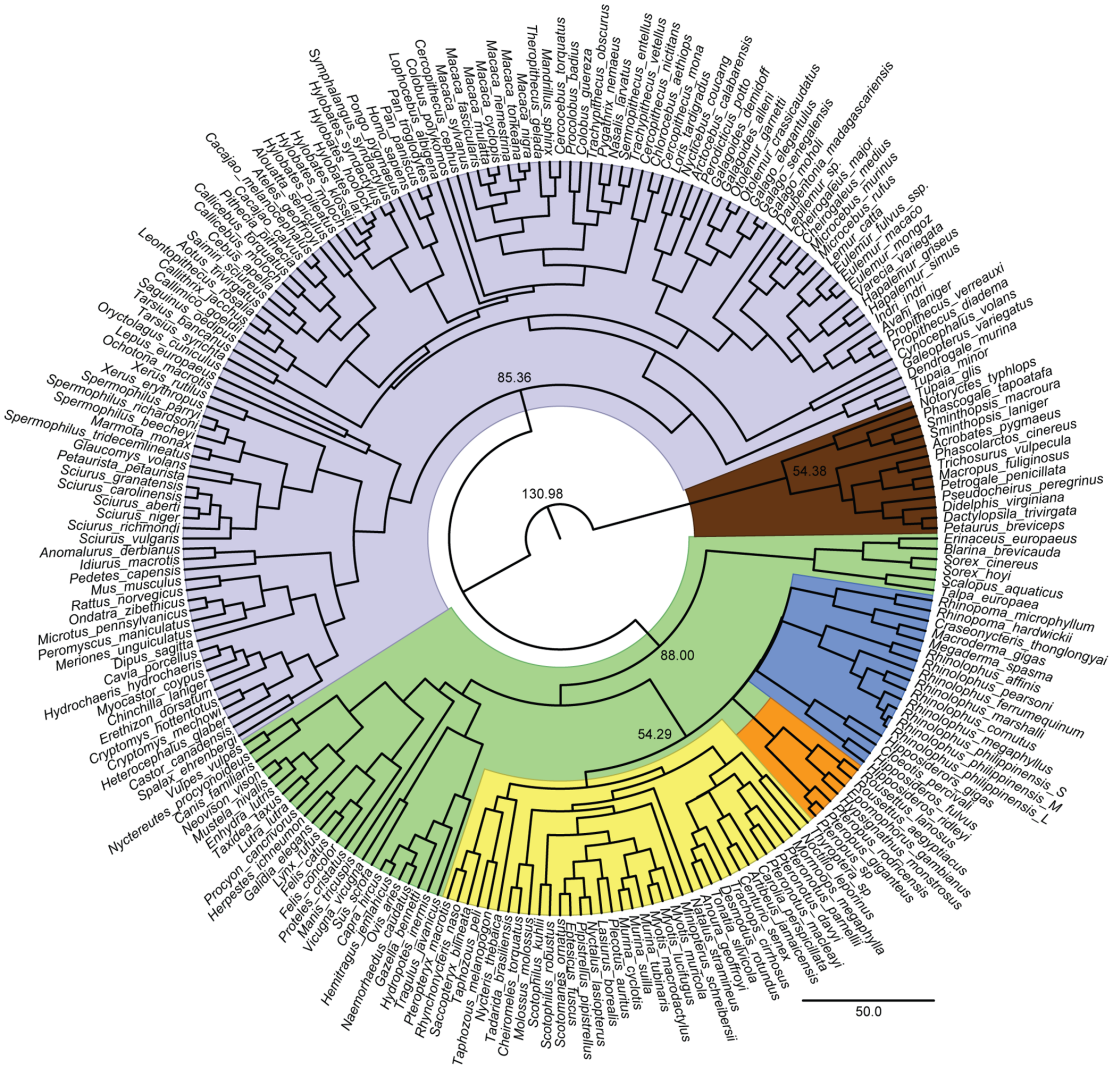
Fossil calibrated *Cytochrome B* species tree. The main clades are coloured as follows: Marsupial mammals (brown); Euarchontoglires (grey); Laurasiatheria (green); Yangochiroptera (yellow); echolocating Yinpterochiroptera (blue); Pteropodidae (orange). The estimated divergence times of Marsupial mammals, Euarchontoglires; Laurasiatheria and Chiroptera are shown on the corresponding nodes.

We studied 68 individuals of 56 bat species from 16 families, with broad taxonomic, geographical and ecological coverage. These included members from both main suborders: the Yinpterochiroptera and Yangochiroptera (see Fig. 1). From the former, taxa from all six families were examined, including five Old World fruit bats (Pteropodidae) that do not use laryngeal echolocation, eight horseshoe (Rhinolophidae) and four roundleaf bats (Hipposideridae) that have evolved CF echolocation, and members of the Megadermatidae, Rhinopomatidae and Craseonycteridae, which all use a range of frequency modulated (FM) echolocation call types. From the latter suborder, ten families were included, all of which also exhibit a range of FM echolocation calls with the exception of *Pteronotus parnellii*, which has convergently evolved CF echolocation [41].

**Figure 2:**
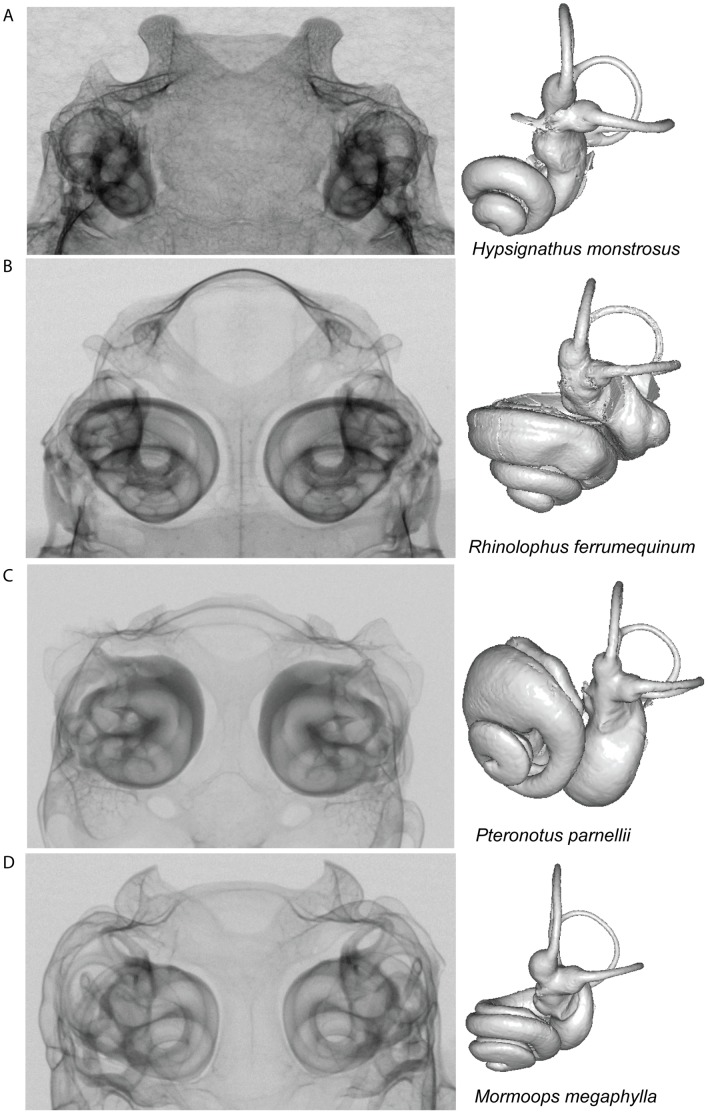
X-ray projections through the posterior portion of bat skulls containing the bony labyrinth, in the ventral-dorsal plane. The corresponding reconstructed inner ear volumes are shown on the right of the panels; these inner ears are orientated so that the lateral semicircular canals are maximally aligned in the horizontal plane, allowing differences in cochlear orientation to be compared across species. Echolocation mode, species and family information are as follows: (A) the non-echolocating Old World fruit bat *Hypsignathus monstrosus* (1.3518) (Pteropodidae); (B) the Old World CF bat *Rhinolophus ferrumequinum* (58.20697) (Rhinolophidae); (C) the New World CF bat *Pteronotus parnellii* (5.21236) (Mormoopidae) and (D) *Mormoops megaphylla* (2.1603) (Mormoopidae) which utilizes narrowband multi-harmonic signals.

Specimens were scanned in the frontal plane using the Metris X-Tek HMX ST 225 CT System at the Department of Mineralogy, EMMA Division, NHM, London. Volumes were reconstructed using CT PRO (Metris X-Tek, UK), and following reconstruction volumes were visualized using VG Studio Max 2.0 (Volume Graphics, Heidelberg, Germany). Internal voids of bony labyrinth were digitally dissected to produce digital endocasts (see Fig. 2 and Fig. S1). The resultant StereoLithography (STL) files, which describe the surface geometry of the volumes, were converted into Stanford polygon files (.ply) with MeshLab v.1.2.2 (MeshLab Visual Computing Lab – ISTI – CNR).

### Measures of Semicircular Canal Size

For each semicircular canal, height and width were measured between canal lumen mid-points, and the radius of curvature (*R*) was calculated as half the average length and width of each canal following Spoor *et al*. [6]. These values were combined with published values for 11 additional bat species [15], [23]. Linear distances were measured directly from each volume surface in Landmark v.3.0 [42].

Before comparing bats to other mammals, we assessed whether the anterior, lateral and posterior semicircular canals showed consistent variation with respect to each other across different bat species. For this, we divided the *R* of each canal within an individual by the cube root (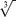) of its body mass, which was obtained either directly from specimen records or from the literature (see Table S1 in Text S1).

#### 1. Semicircular canal size in bats versus non-flying mammals

We tested the prediction that bats have proportionally larger semicircular canals than non-flying mammals by comparing our bat *R* values with published values from 156 non-bat species [15], [17], [23]. Where multiple specimens per species were available, linear measurements were averaged prior to analysis, with the exception of the three *R. philippinensis* size morphs which are treated as independent taxa in all analyses. Radius of curvature values and 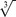
body mass values were log_10_ transformed to normalise the variance. To explore the allometric relationship between log semicircular canal size and log 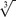
body mass, we used the modified protocol of Knell [43]. Here we tested whether the data followed a simple linear relationship across all taxa, or whether – due to the wide variation in mammalian body size – they were better described by a discontinuous model (see supplementary methods and results in Text S1). These analyses were undertaken in Rv.2.11.1 [44] and repeated using phylogenetic information in MCMCglmm (see below). To test whether canal size has been influenced by the evolution of flight and laryngeal echolocation, we also assessed model fit after adding these variables, while accounting for the phylogenetic relatedness of species.

#### 2. Relationship between semicircular canal morphology and cochlea size

To assess how the evolution of the vestibular system within echolocating bats has been influenced by the expansion of the cochlea, we used two approaches. First, we examined the relationship between relative semicircular canal size and relative cochlea size. We calculated cochlea size as the average of the diameter of the first cochlear turn, the second cochlear turn, and the “slant height” following Spoor *et al.* [17], thus allowing us to compare our results with published labyrinth values from 40 non-bat placental and marsupial mammals [17]. Log relative semicircular canal *R* was plotted against log relative cochlea size for the non-bat mammals, and Ordinary Least Squares (OLS) and Reduced Major Axis (RMA) regressions were calculated in PAST v.2.0 [45].

Second, we quantified canal shape using the geometric morphometric technique of eigenshape analysis [46], [47], and then related changes in canal shape to relative cochlea size. Canal shape is likely to be important in determining the flow of fluid through the semicircular canal lumens. Shape variation of each approximately planar semicircular canal was captured with a 2D outline along the internal edge using Image-Pro Plus v.5.1 (Media Cybernetics Inc., Bethesda, MD, USA) (see Fig. S2). The initial outlines of 200 equally-spaced semi-landmarks were subsequently down-sampled to 100 points for analysis using FORTRAN routines written by N. MacLeod [48]. Models were constructed to visualise shape change along each axis (see supplementary methods in Text S1). For shape analyses one representative per species was used. To summarise overall semicircular canal shape variation across bats a canonical variates analysis (CVA) was performed on the eigenscores that explained 95% of the sample shape variance (see supplementary methods and results in Text S1).

#### 3. Relationship between semicircular canal size and wing morphology

To test whether semicircular canal size correlates with flight performance, we used three wing parameters that are considered important determinants of aerobatic manoeuvrability: (a) wing aspect ratio (WAR) and (b) wing tip shape index (WTS), which both describe wing shape, and (c) wing loading (WL), which measures the force per unit area of the wing [32]. Bats characterised by low WAR are expected to have short broad wings, which combined with low WL, will typically confer slow and manoeuvrable flight suited to high clutter environments [49]. For the final measurement, WTS, higher values mean a blunt round ended wing whereas low values imply a more pointed wing [50]. These values were obtained either directly from wing traces and photographs or from published sources (see Table S1 in Text S1 for values and sources), and were log_10_ transformed prior to analyses. The effects of wing morphology and body mass on semicircular canal size were examined using mixed effect models that account for phylogeny (described below). These analyses were applied independently to three different sets: (i) all bats, (ii) members of suborder Yangochiroptera, and (iii) echolocating members of the suborder Yinpterochiroptera following Teeling *et al.* [34].

### Phylogeny Construction and Bayesian Phylogenetic Mixed Models

To control for the shared ancestry of morphological characters, the above analyses were undertaken using Bayesian phylogenetic mixed models (BPMMs) implemented in ‘MCMCglmm’ [51] in R v.2.11.1, which have been developed specifically for this purpose (see supplementary methods in Text S1). This model relies on an accurate phylogeny, and therefore *Cytochrome b* sequences were obtained for as many species as possible, mainly from GenBank. For a few species where sequences were not available, we either used data from a congeneric taxon where available, or excluded the species from this analysis. Where multiple haplotypes were available, we selected a representative sequence arbitrarily. Nucleotide sequences were aligned using ClustalW2 [52] and checked by eye. The alignment was imported into BEAUti v.1.5.4, which was used to produce the correctly formatted input file (xml-file) for BEAST v.1.5.4 [53]. The topology was constrained by enforcing monophyly of major clades, together with a total of 16 fossil calibration points collected from several literature sources: split of placental and marsupial mammals 131.0 Ma [54]; Carnivores 57.5 Ma [54]; Primates 58.5 Ma [55]; Artiodactyla 60.0 Ma [56]; Leporidae 53.0 Ma [57]; Dermoptera 35.5 Ma [58]; base of Felidae 16.0 Ma [59]; split of Lorisidae and Galagidae 41.2–36.9 Ma [60]; split of *Mus* and *Rattus* 12.0–14.0 Ma (references within [61]). Bat fossil calibrations included the oldest fossil bat dated at 52.5 Ma [36]; a maximum for base of Rhinolophoidea 55.0 Ma, minimum for base of Emballonuridae 37.0 Ma, minimum for base of Rhinolophidae 37.0 Ma, minimum for split of Mormoopidae and Phyllostomidae 30.0 Ma, maximum for base of Phyllostomidae 34.0 Ma and minimum for split of Vespertilionidae and Molossidae 37.0 Ma (following [62]). Despite recent advances in molecular phylogenetics, the exact placement of the Chiroptera within the Laurasiatheria remains contentious [63]–[65]. For this study, bats were treated as the sister group to Cetartiodactyla, Carnivora and Perissodactyla, consistent with recent studies [66], [67]. Analyses were run in BEAST v.1.5.4 using an uncorrelated log-normal relaxed molecular clock [68], Yule speciation prior, GTR+I+Γ model, for 10,000,000 generations, with every 1000 parameters logged. Calibration points were set with a normal prior distribution with ±0.5 standard deviation. Tracer v.1.5 was used to check for run convergence and appropriate burn-in length. The maximum clade credibility tree was produced using TreeAnnotator v.1.5.4, with a sample burn-in of 200 and node heights set to mean-heights (see Fig. 1). For the calibrated phylogeny see the Dryad Repository (http://dx.doi.org/10.5061/dryad.r0789).

To compare models utilising phylogenetic information, we examined the Deviance Information Criterion (DIC); with ΔDIC values ≥2 taken to indicate significantly improved model fit. For tests of fixed effects, we report the *P*_MCMC_ value, which is twice the posterior probability that a model parameter is greater or less than zero (whichever is lower), as estimated by the Markov chain, and is one possible Bayesian analogue to a two-tailed frequentist p-value.

## Results

### 1. Semicircular Canal Size in Bats Versus Non-flying Mammals

For each semicircular canal, we investigated the allometric scaling of *R* versus body mass after accounting for the potentially confounding effects of phylogeny (see supplementary information in Text S1 for full results). All model comparisons suggested that a negative allometric linear relationship with two size classes resulted in improved DIC values across a sample including both flying and non-flying mammals (ΔDIC = 7.31, 12.75 and 3.94 for anterior, lateral and posterior, respectively; see Table S2C in Text S1). However, models that included either flight or laryngeal echolocation did not result in improved model fit (respective anterior ΔDIC = −0.19 and −0.64; lateral ΔDIC = 0.13 and 0.09 and posterior ΔDIC = −0.27 and −0.48; also see Table S2C in Text S1). Therefore after accounting for the non-independence of species, no effect of either trait on semicircular canal size was found and thus there is no evidence to suggest that bats consistently display larger semicircular canals compared to non-flying mammals.

**Figure 3:**
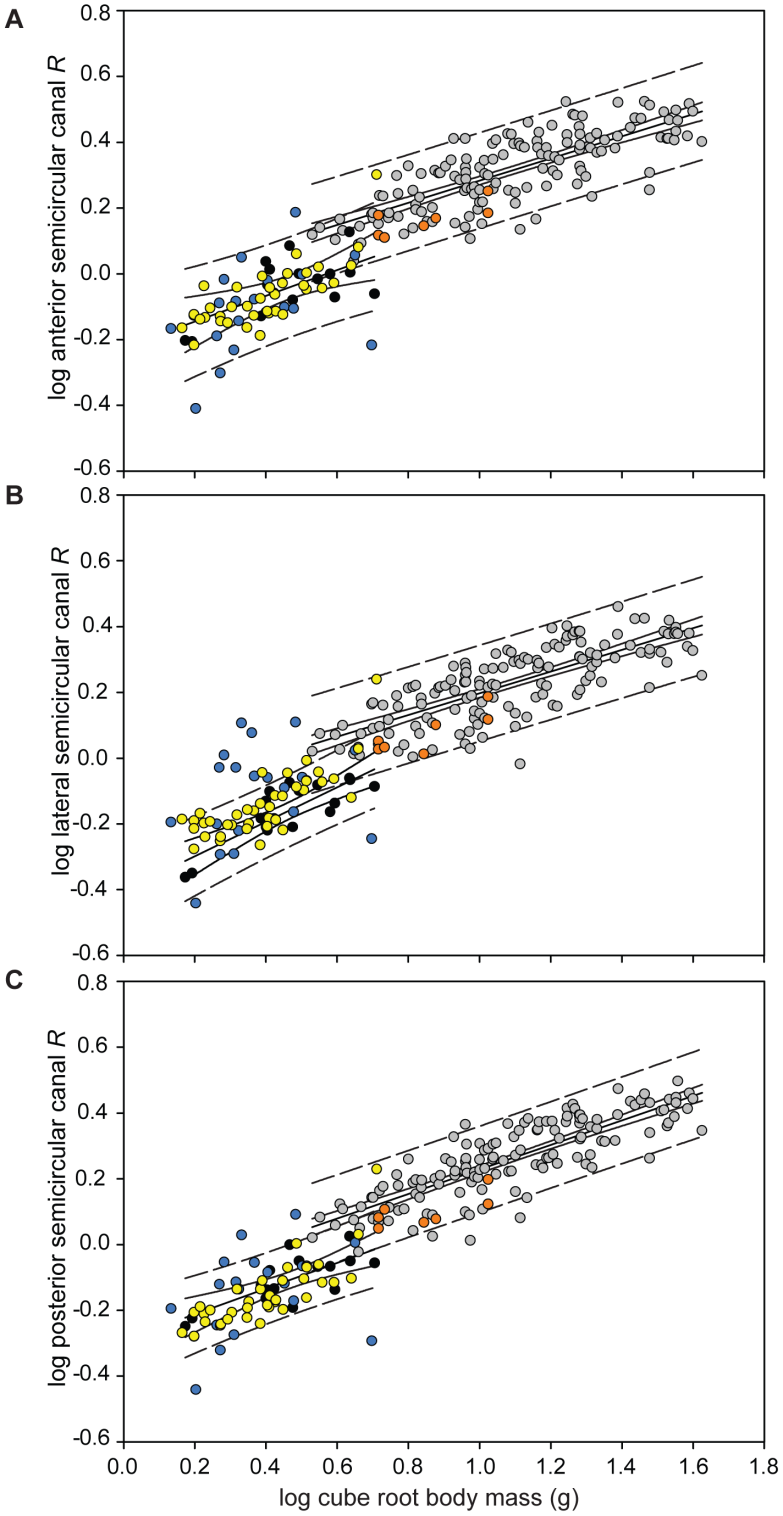
Log semicircular canal radius (*R*) versus log body mass^0.33^– for (A) anterior, (B) lateral and (C) posterior semicircular canals across bats [Old World fruit bats (orange); echolocating Yinpterochiroptera (blue); Yangochiroptera (yellow)] and non-flying mammals [species designated as small body-sized (black) and species designated as large body-sized (grey) according to the data]. 95% prediction intervals (dashed lines) and 95% confidence intervals (solid lines) are shown for the mammal regression lines. Lateral semicircular canal size of echolocating bats showed the most variation of all three canals; species falling above the 95% PI (and thus larger lateral canals than expected) were six *Rhinolophus spp*., *Rhinopoma hardwickii*, *Cardioderma cor* and *Pteronotus parnellii*. Across all three canals, species with consistently larger canals included *C. cor* and *Rhinolophus megaphyllus* and those with consistently smaller were *Cloeotis percivali, Rhinolophus philippinensis* (small morph) and *Macroderma gigas.* However, it should be noted that OLS regression prediction and confidence intervals calculated assume independent data points and, therefore, the intervals presented here may underestimate the actual values.

**Figure 4:**
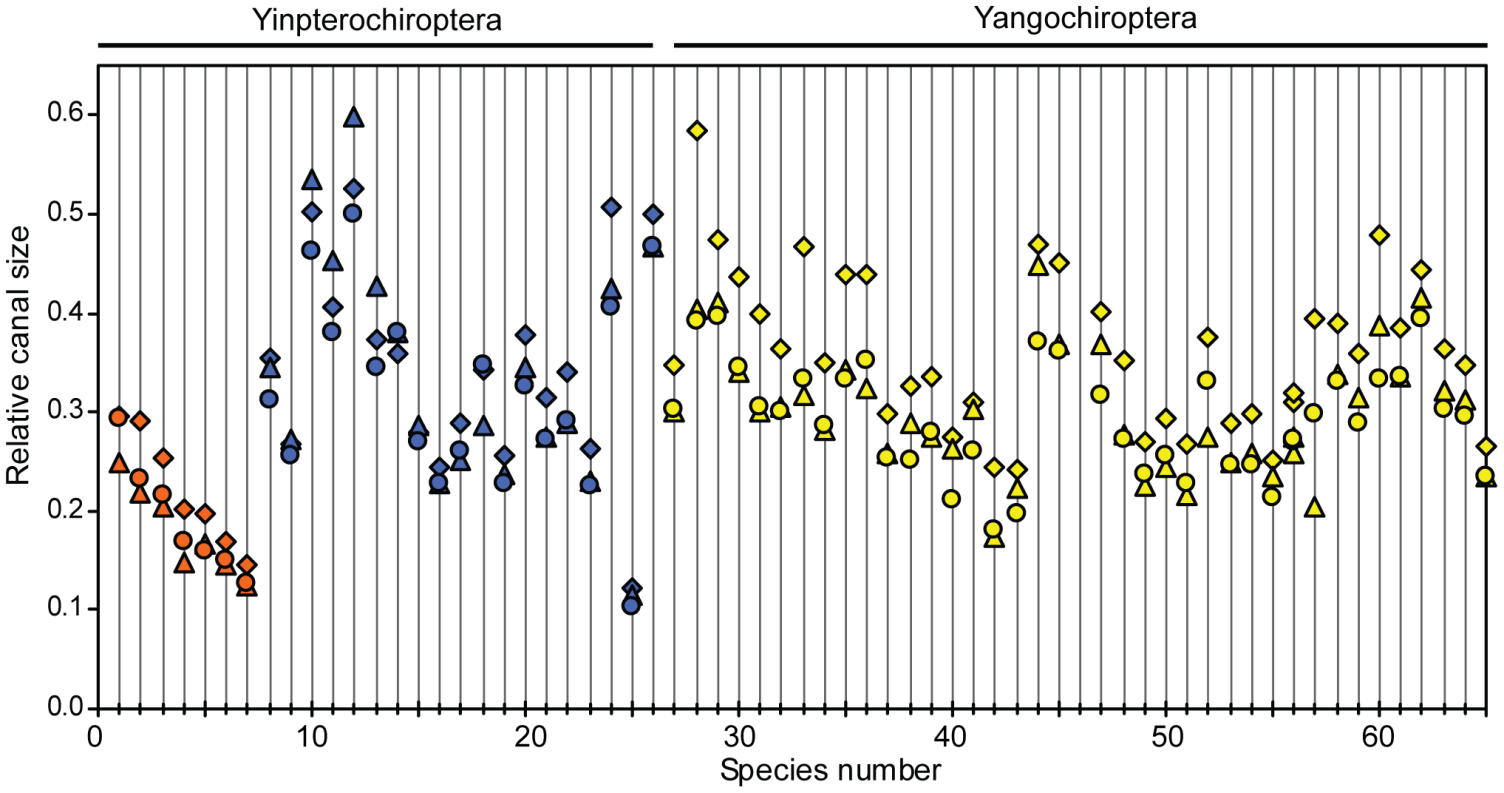
Relative size of the three semicircular canals within bats. The three canals are depicted as follows: anterior – diamonds; lateral – triangles and posterior – circles. With members of the two bat clades represented as follows: Yinpterochiroptera [Old World fruit bats (orange), laryngeal echolocating species (blue)] and the Yangochiroptera (yellow). Species numbers follow those in Table S1 in Text S1.

In support of this, although plotted values of log semicircular canal size versus log 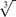
body mass revealed considerable variation in bat relative canal size (Fig. 3), echolocating bats from both suborders typically fitted the distribution expected for their body mass, with most species within the 95% prediction intervals (PI) of the anterior and posterior semicircular canals (Fig. 3A and C). Values for all three semicircular canals of non-echolocating Old World fruit bats fell below the regression line for their ‘size class’ (see supplementary results in Text S1 for explanation) but remained within the 95% PIs. A significant correlation was found between canal radius and body mass in Old World fruit bats in two canals (anterior: *r = *0.76, T statistic = 2.65, *P = *0.045; lateral: *r = *0.83, T statistic = 3.35, *P = *0.020; posterior: *r = *0.72, T statistic = 2.32, *P = *0.068, all with DF = 5). Similarly in the Yangochiroptera all three canals were found to positively correlate with body mass (anterior: *r = *0.76, T statistic = 7.02, *P = *3.647×10^−8^; lateral: *r = *0.78, T statistic = 7.44, *P = *8.645×10^−9^; posterior: *r = *0.81, T statistic = 7.39, *P = *1.208×10^−8^, with DF = 35, 36 and 35, respectively). In echolocating Yinpterochiroptera no correlation was found between canal radius and body mass (anterior: *r = *0.44, T statistic = 1.99, *P = *0.062; lateral: *r = *0.28, T statistic = 1.26, *P = *0.225; posterior: *r = *0.35, T statistic = 1.52, *P = *0.148, with DF = 17, 18 and 17, respectively). Furthermore, relative sizes of the three semicircular canals differed widely among bat species (see Fig. 4): in all Yangochiroptera and Old World fruit bats, the anterior canal was the largest, whereas in horseshoe bats, it was the lateral canal that was typically largest. In most bat species, the lateral and posterior canals were similarly sized to each other, again with the exception of horseshoe bats in which the posterior canal was smaller.

### 2. Relationship between Semicircular Canal Size and Morphology and Cochlea Size

**Figure 5:**
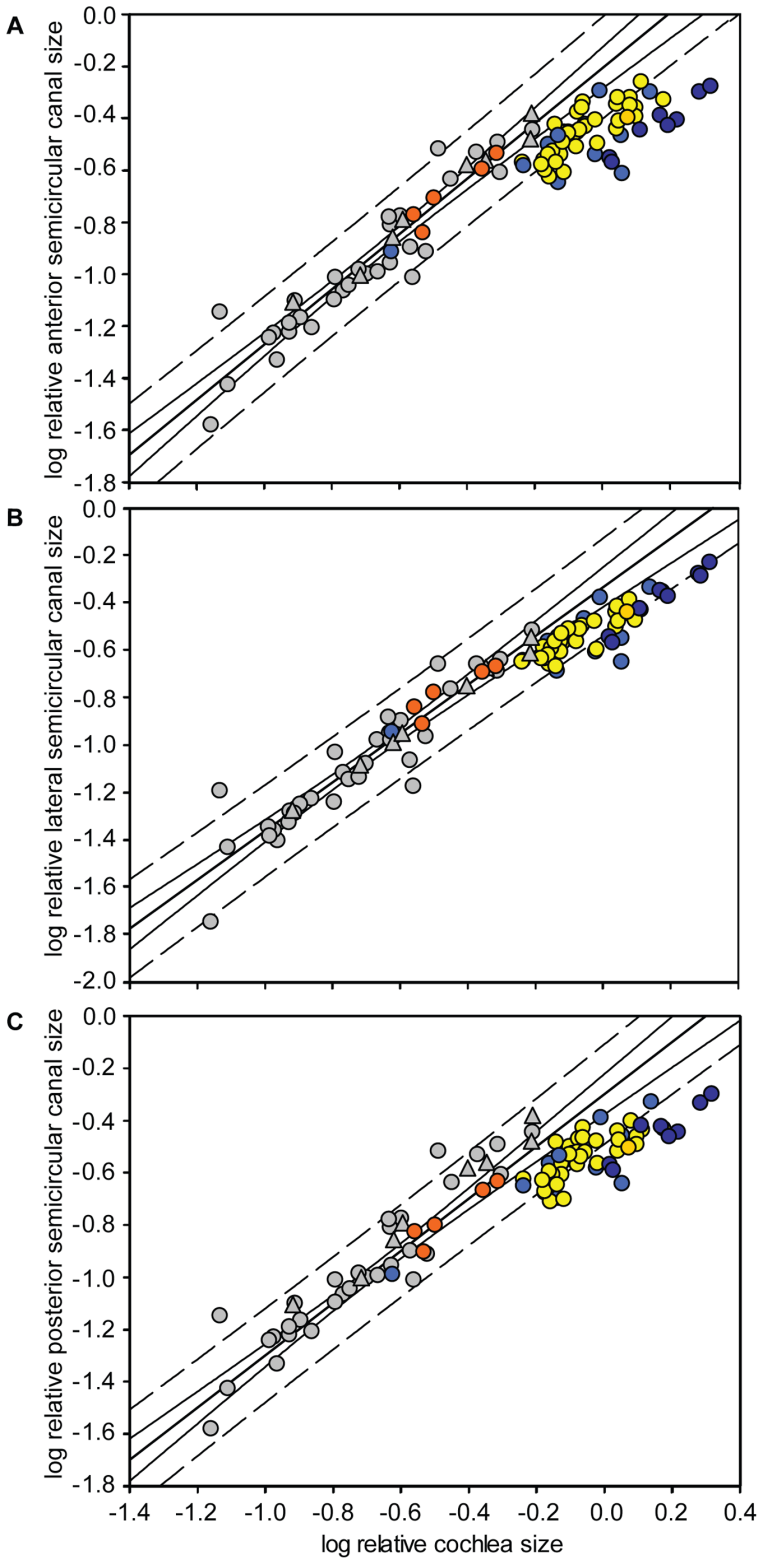
Relative (A) anterior, (B) lateral and (C) posterior semicircular canal size versus relative cochlea size. The OLS regression of non-bat mammals is shown by the bold line (anterior: *r = *0.954, *P* = 3.48×10^−20^; lateral *r* = 0.946, *P* = 1.91×10^−21^; posterior *r* = 0.951, *P* = 7.29×10^−21^), and the 95% confidence and prediction intervals are shown by the solid and dashed lines respectively. Non-bat placental mammals (grey circles) and marsupial mammals (grey triangles) from Spoor *et al*. [17]. Pteropodidae (orange circles), Yangochiroptera (yellow circles), *Pteronotus parnellii* (gold circle), Rhinolophidae (dark blue circles), and remaining echolocating Yinpterochiroptera (medium blue circles) represent the bat species included by this study.

Log relative semicircular canal *R* was plotted against log relative cochlea size for all bat species and 40 non-bat placental and marsupial mammals. Ordinary least square regression lines with 95% confidence and prediction intervals were fitted to the non-bat data and the bat values superimposed (Fig. 5). Ordinary least square and RMA regression of the 40 non-bat species revealed slopes ∼1 i.e. isometry (OLS slope: anterior 1.07; lateral 1.03; posterior 1.00; RMA slope: anterior 1.12; lateral 1.09; posterior 1.06). Among the bats, all three semicircular canals of members of the Pteropodidae and Megadermatidae fell within the 95% PI, whereas in the other echolocating bats, points for all three canals fell below the line of best fit, but usually within the 95% PIs. The main exceptions were horseshoe bats, roundleaf bats and *P. parnellii,* all of which use CF echolocation (also see Fig. 2B and C). For these taxa, the anterior and posterior canals were small for their cochlea size, falling below the 95% PIs (Fig. 5A and C, respectively). This association between CF echolocation and relatively small anterior and posterior canal sizes was confirmed using phylogenetic mixed models, with CF echolocation fitted as a factor (ΔDIC: anterior: 2.50; posterior: 3.27; lateral: 0.58; see Table S3 in Text S1).

**Figure 6:**
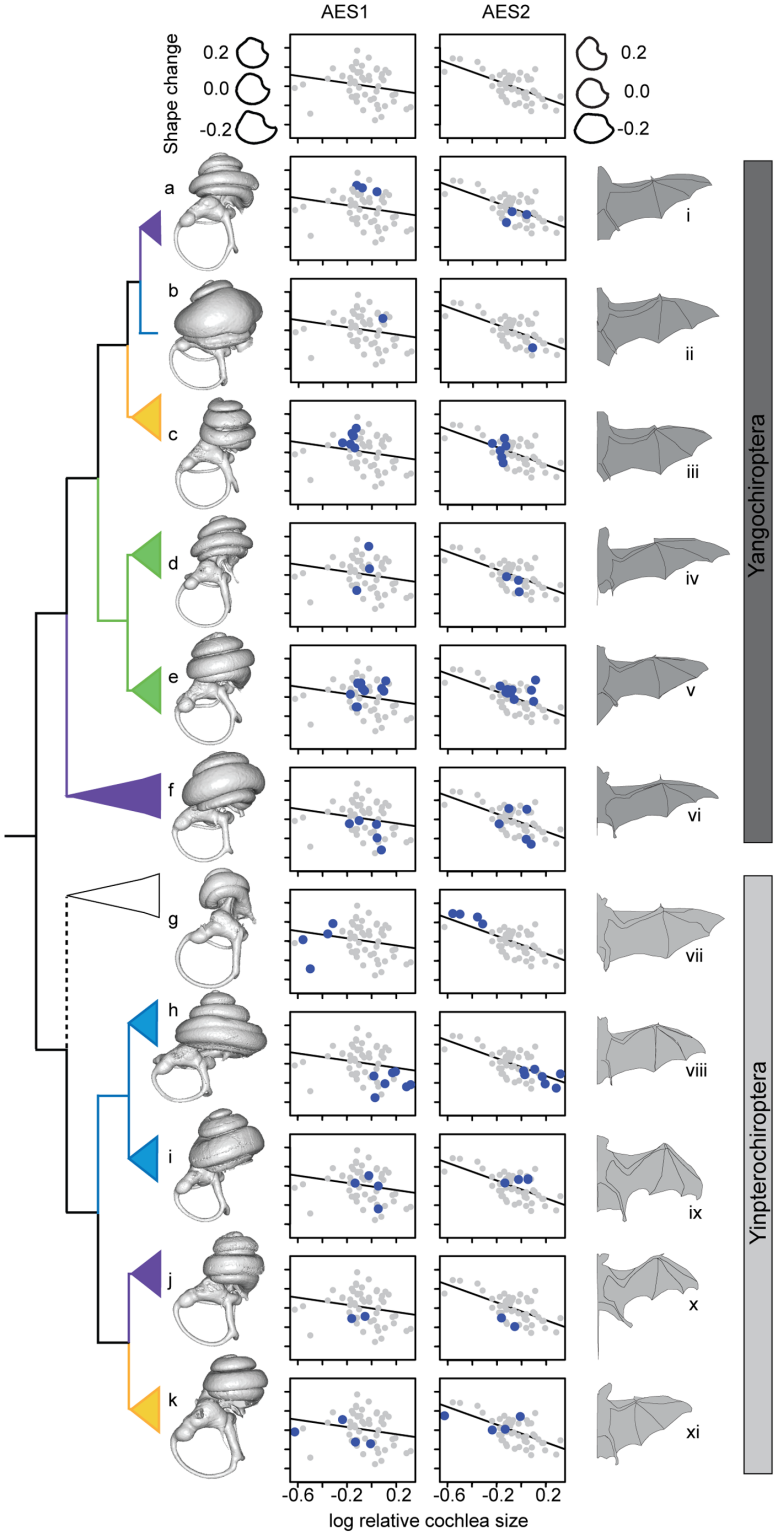
A sample of the bat families studied with ecomorphological characters mapped onto the phylogeny. Bat phylogeny to show species representation based on Teeling *et al.* [34]. Family call types are taken from Jones and Teeling [41]; no laryngeal echolocation (white); short, broadband, multiharmonic (yellow); narrowband, multiharmonic (purple); constant frequency (blue); narrowband, dominated by fundamental harmonic (green). Labyrinths obtained from this study include Yangochiroptera (dark grey bar): *Mormoops megalophylla* (a), *Pteronotus parnellii* (b), *Trachops cirrhosus* (c), *Cheiromeles torquatus* (d), *Lasiurus borealis* (e) and *Saccopteryx bilineata* (f); Yinpterochiroptera (light grey bar): *Rousettus aegyptiacus* (g), *Rhinolophus pearsonii* (h), *Hipposideros ridleyi* (i), *Rhinopoma hardwickii* (j) and *Lavia frons* (k). Representative wing outlines, traced from published figures, as follows: *Morpmoops blainvillii* (i) (modified from [86]); *Pteronotus parnellii portoricensis* (ii) (modified from [86]); *Artibeus jamaicensis jamaicensis* (iii) (modified from [86]); *Tadarida pumila* (iv) (modified from [49]); *Pipistrellus pipistrellus* (v) (modified from [49]); *Taphozous melanopogon* (vi) (modified from [87]); *Eidolon helvum* (vii) (modified from [49]); *Rhinolophus ferrumequinum* (viii) (modified from [88]); *Hipposideros abae* (ix) (modified from [89]); *Rhinopoma hardwickii* (x) (modified from [89]); *Lavia frons* (xi) (modified from [49]). Plots represent family distributions along shape axes of the anterior semicircular canal eigenshape 1 (AES1) and anterior semicircular canal eigenshape 2 (AES2).

The linear relationships between semicircular canal shape (as quantified by eigenshape analysis) and relative cochlea size are shown in Fig. 6 and Fig. S3A (see Table S4A in Text S1 for regression statistics). Examination of shape change shown by the models suggested that the canals of bats with large cochleae showed greater deviations from circularity (Fig. 6 and Fig. S3A). To determine whether such changes in canal shape with increased relative cochlea size were supported statistically while controlling for phylogeny we used mixed models; here, eigenshape axes describing canal shape were fitted alongside log 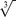
body mass and then the model performance was again assessed after adding log cochlea size. We recorded improved fit for the first two eigenshape axes describing anterior semicircular canal shape (ΔDIC = 8.67, 2.14 and −1.57, for ES1, 2 and 3, respectively; see Table S4B in Text S1). None of the corresponding mixed models, for either lateral (ΔDIC = 0.21, −0.08 and −5.17, for ES1, 2 and 3 respectively) or posterior semicircular canals (ΔDIC = −2.83, −13.64 and −4.51, for ES1, 2 and 3 respectively) improved model fit (see Table S4B in Text S1). Interestingly, of the three axes describing lateral semicircular canal shape, ES2 and ES3 showed a significant relationship with log 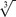
body mass: *P*_MCMC_ *= *0.012 and 2.22×10^−4^ for ES2 and ES3 respectively, whereas ES1 was not significant; *P*_MCMC_ *= *0.058. Canonical variates analysis (CVA) of the anterior semicircular canal eigenscores suggest that Old World fruit bats and echolocating bats, from both main suborders, are clearly separated (Wilks’ λ = 0.148; DF = 40, 66; *P*<0.001), with minimal overlap between the two groups of echolocating bats (Fig. S3B). The CVA of lateral semicircular canals again showed a clear separation of the Old World fruit bats from the remaining bats, but with a larger overlap between echolocating species. Again, this separation was significant (Wilks’ λ = 0.225; DF = 40, 72; *P = *0.005). The posterior semicircular canal CVA showed minimal sample overlap of all the two echolocating groups, however, the grouping was significant (Wilks’ λ = 0.210; DF = 40, 66, *P = *0.008) (see Fig. S3B).

### 3. Semicircular Canal Size and Flight Manoeuvrability Inferred from Wing Morphology

We first explored the relationship between semicircular canal *R*, body mass and wing morphology using mixed models across all bat species examined. For each of the three canals, only log 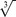
body mass was found to have a significant relationship with log semicircular canal *R* (*P*_MCMC_ <1×10^−4^ in all cases, Table S5A in Text S1). Furthermore the sequential addition of both wing parameters, wing loading (WL) and wing aspect ratio (WAR), did not improve model fit; for anterior ΔDIC = −1.69 and −1.73, respectively, for lateral ΔDIC = −1.34 and −1.69, respectively, and for posterior ΔDIC = −1.49 and −0.63, respectively; see Table S5A in Text S1). Analysis of each group of echolocating bats (i.e. Yangochiroptera and some members of the Yinpterochiroptera) also showed no effect of WL and WAR, however, in the former group log 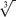
body mass was a significant determining factor for all three canals (*P*_MCMC_ <1×10^−4^ in all cases, Tables S5B and C in Text S1).

We also found no overall significant effect of wing-tip shape WTS on semicircular canal size (Table S5A in Text S1), however, plots of log *R* versus WTS for each echolocating suborder revealed a possible outlier within echolocating Yinpterochiroptera – *Rhinolophus philippinensis* (small morph) (Fig. S4). After excluding this taxon – which appears to have evolved recently via a large shift in echolocation call frequency [69] – we found a positive significant relationship with canal size in all three canals, indicating that canal *R* increased with wing roundedness (anterior *P*_MCMC_ *= *0.024, lateral *P*_MCMC_ *= *0.009, posterior *P*_MCMC_ = 0.025; summarised in Fig. S4).

## Discussion

We undertook micro-CT scans of the inner ears of a wide range of bats, and used phylogenetic models of trait evolution to test whether the mammalian vestibular apparatus has been influenced by the evolution of powered flight and laryngeal echolocation. Initial analyses revealed evidence of differential scaling between body mass and semicircular canal size in large and small-bodied mammal species, although the allometric relationship was of the same order in each case (see also [70], [71]). Semicircular canal mechanics, and therefore sensitivity, depend on many factors, including canal length, lumen radii and relative arrangement of canals (e.g. [21], [29]). These relationships suggest that multiple factors can be fine-tuned in order to optimize canal sensitivity. For example, it has been hypothesized that in very small bodied animals, head size will limit canal size [29] and, therefore, it is possible that the vestibular systems of these species have seen modifications such as increases in canal lumen width to counteract this. This is one possible explanation for the observed pattern that mammals with low body mass have proportionally smaller canals than those with greater mass, even allowing for the negative allometric relationship, although additional comparative studies are needed to test this. Bats showed similar trends to non-flying mammals of similar body size; therefore, while they are likely to face unique challenges to maintain a sense of balance during flight we found no effect of powered flight *per se* on semicircular canal size.

The absence of a detectable effect of powered flight on semicircular canal size in bats contradicts our predictions based on birds [22]; in particular that birds have enlarged semicircular canals compared to mammals and dinosaurs [13], [22], and also that bird species capable of higher aerial mobility tend to have longer and narrower canals [26]. Although enlarged semicircular canals have also been documented in a third group of flying vertebrates – the pterosaurs – this probably reflects their reliance on vision during hunting and the associated expansion of the flocculus, rather than aerial agility [11]. Such contrasting results might reflect differential physiological constraints acting on phylogenetically disparate groups; for example, birds have undergone more dramatic physical modifications for flight, and might also isolate their visual and vestibular systems from body movements during locomotion (e.g. accelerations due to linear and angular displacement) [72]. Indeed, gliding mammals have also been shown not to possess markedly enlarged vestibular systems, for example when compared with arboreal taxa (see figure 1 from [15]).

We also hypothesized that the semicircular canals of echolocating bats would show additional deviations in size caused by hypertrophy of the cochlea. In support of this, the horseshoe bats (Rhinolophidae) – and to a lesser extent echolocating bats in general – had smaller canals relative to their cochlea size. Echolocating bats were also found to display greater inter-specific variation in canal size compared to non-flying mammals. Such variation may reflect the wide spectrum of hearing frequency ranges and cochlea sizes seen in bats, although we cannot rule out the possibility that they might also reflect the extent to which different taxa rely on vision. Apart from these overall trends, we also found that the three canals varied in relative size with respect to each other across taxa. For example, the horseshoe bats had proportionally large lateral canals for their body mass, which were also the largest of the three canals in this group, whereas in most mammals (including other bats measured here) it is the anterior canal that is largest (as summarised in [23]).

As well as influencing canal size, eigenshape analyses revealed that body and cochlea size also impacted on canal shape, with more elliptical canals recorded in smaller species or in those with the largest relative cochleae. Most notably, the horseshoe bats – which have the largest cochleae, relative to body mass (also see Fig. 2) – possessed the least circular (most elliptical) canals. Interestingly, although the effects of body size on shape were seen to be most obvious in the lateral semicircular canal and the impact of relative cochlea size was greatest in the anterior canal, in both cases both morphological parameters are likely to be inter-related. For example, a comparison of the skulls of non-echolocating Old World fruit bats (Fig. S1A) and laryngeal echolocating bats (Fig. S1B–D) suggested that the region containing the vestibular system in echolocating taxa is displaced outwards relative to the midline of the skull, possibly related to the expansion of cochlea within the skull of these small bodied animals. However, in echolocating species, the lateral semicircular canal may to some extent be ‘shielded’ from any spatial constraint by the basal expansion of the cochlea (such that the most distal point of the lateral canal of the horseshoe bat does not project further than that of the cochlea; see Figs. 2B and 5B). In contrast, the anterior semicircular canal projects directly away from the cochlea in most species thus a more direct conflict might be expected (e.g. Fig. 2D, but see Fig. 2C). It follows that each canal appears to be under different selection pressures depending on its orientation and position within the petrosal bone, a possibility that could also help to explain the contrasting patterns of inter-canal size variation within individuals from different taxa (e.g. [12]). Although suggestions that small body mass should correlate with structural constraints in the semicircular canals have been made before, they have hitherto received little supporting evidence [23], [73]. Our findings therefore suggest that the hypertrophic cochleae of echolocating bats, combined with their small body size, could create more extreme spatial pressures on the vestibular system than in similarly sized non-echolocating mammals. Furthermore, echolocating bats all show highly modified basal crania with an articulation between the stylohyal and the tympanic bone [74], [75], which might impose additional spatial constraints on the vestibular apparatus.

The link between semicircular canal shape and sensitivity is currently poorly understood, although elliptical canals may be less sensitive than circular ones [20]. At the same time, however, it has been predicted that only extreme deviations from circularity will significantly reduce sensitivity, and that where such deviations occur, they might be counteracted by an increased internal lumen radius [20], [23]. Consequently, very small animals may have proportionally wider semicircular ducts [20] and lumen duct radii would thus represent important additional morphological parameters of bat vestibular systems that warrant future study.

In bats, wing morphology and flight performance correlate well with aspects of echolocation call structure, all of which define the ecomorphological niche [50]. Consistent with this idea, we found some limited evidence that semicircular canal size in most echolocating members of the Yinpterochiroptera correlates positively with the roundness of the wing (wing tip index). Thus the size of the semicircular canals in this clade might be adapted to their characteristic slow and manoeuvrable flight, or alternatively, could be an artefact of their enlarged cochleae. One notable outlier in our analyses was *Rhinolophus philippinensis* (small morph), which was found to possess smaller inner ear structures (including the semicircular canals) than its relatives. This result was of particular interest because this taxon appears to have a recent origin, probably evolving rapidly via a shift in call frequency that might also have involved a change in its inner ear dimensions [69].

In the other major group of echolocating bats – the Yangochiroptera – canal size showed no association with wing parameters, but did correlate with body mass alone. The absence of any stronger relationships between canal and wing morphology in both suborders of bats could also reflect limitations of the wing descriptors most commonly available. Indeed, although wing morphology has been commonly related to flight and ecological characteristics (e.g. [32], [76], [77]), the metrics used to describe vertebrate wings (e.g. WAR, WL and WTS) are borrowed from the field of aerodynamics. As such they might poorly apply to the membranous wings of bats for which stationary wing measurements likely differ from the true wing surface-area during flight [78]. Furthermore, in the case of our study, other sources of variation might also obscure any real patterns, such as potential shrinkage of wing membranes from museum specimen preservation, the use of published data collected by different individuals, and the potential for intra-specific variation and seasonal fluctuation in body mass that will influence estimates of wing loading.

In addition to exhibiting inter-specific variation in echolocation call type and flight performance, bats also show other sensory, anatomical and ecological differences that could exert selection pressures on their vestibular systems. Many of these factors could not be tested here due to a paucity of comparative data. For example, species differ in their roosting postures [79] and associated movements [80] and also vary in their capacity for terrestrial locomotion [81], [82]. In terms of sensory modalities, some bats emit echolocation calls nasally, and others orally, each of which requires different head orientations that in turn affects the orientation of the lateral semicircular canal [83]. Head movements resulting from compensatory cervical reflexes are also crucial for stabilisation of gaze [6], and morphological convergence in the cervical vertebrae of echolocating bats has previously been documented [79]. Finally, as previously stated, dependence on vision (as summarised in [84]) as well as orbital convergence and musculature position (e.g. [8]) all vary among bat species and so might also be linked to canal morphology given the tight link between these systems as exemplified by the vestibulo-ocular reflex.

Recent studies concerning the geometric arrangement of the semicircular canals in primates concluded that angular deviation from the orthogonal arrangement of 90° is directly related to the speed of head rotations [10]. This evidence, as well as similar findings from other studies, might also be applicable to other taxonomic groups [10], [16], so potentially explaining reported taxon-specific inconsistencies to the orthogonal canal arrangement (e.g. [85]). The lack of correlation between measures of canal *R* and recorded angular head rotations is also noteworthy, as this may call into question methods previously used to quantify agility [10]. Furthermore, it was suggested that differential demands may dominate the vestibular system in species experiencing either high or low angular velocities [10]. Given the lack of strong evidence supporting a correlation between measured *R* values and flight in bats, this approach may help to increase our understanding of the evolution of bat vestibular systems.

To conclude, our results provide good evidence that semicircular canal morphology has undergone specialisations linked to unique evolutionary innovations seen in bats. Although we found no evidence that bat semicircular canal size has been affected by the acquisition of powered flight, we did find an effect of cochlea size on both canal size and shape. Strikingly, while the vestibular systems of all laryngeal echolocating bats seemed to display similarly modified semicircular canals, possibly indicating similar spatial constraints, the canals of non-echolocating Old World fruit bats appeared to be small for their body size and in proportion to the relative cochlea size. At the same time however, species from the two evolutionary distinct groups of echolocating bats displayed significantly different semicircular canal morphologies in terms of shape and allometrical scaling. Similar inner ear dimensions seen in Old World fruit bats and other non-echolocating mammals, with no deviations from circularity detected, do not support the theory that Old World fruits bats once possessed the hypertrophic cochlea characteristic of echolocating bats. Consequently, of the two main scenarios proposed to explain the paraphyly of echolocating bats, our data are most consistent with the convergent evolution of echolocation in the Yangochiroptera and Yinpterochiroptera suborders rather than its loss in Old World fruit bats.

## Supporting Information

Figure S1 **Micro-computed tomography scan slice through four bat skulls, displaying the relative position of the three semicircular canals within the skull.** Scans are from the following species: (A) *Pteropus rodricensis* (BMNH.76.3.15.14); (B) *Myotis lucifugus* (BMNH.7.7.7.3359); (C) *Rhinolophus ferrumequinum* (58.20697) and (D) *Cloeotis percivali* (BMNH.66.5456). Abbreviations: ASC – anterior semicircular canal; LSC – lateral semicircular canal; PSC – posterior semicircular canal.
(TIF)

Figure S2 **A Reconstructed inner ear volume of** ***Trachops cirrhosus*** **(BMNH.1924.3.1.33) indicating the orientation of each semicircular canal for outline collection.** Starting points (white arrows) for each canal outline (red lines) were as follows: anterior semicircular canal (top left) - point of inflection of the ampullae; posterior semicircular canal (top right) - maximum point of curvature at apex of canal; lateral semicircular canal (bottom panel) - where canal projects freely from the base. **B Mean sample shapes for (left – right) anterior, posterior and lateral semicircular canals.** Outlines represent the mean semicircular canal shape of the morphological variation of 55, 54 and 58 individuals respectively, and are represented by 100 coordinate points.
(TIF)

Figure S3 **A Semicircular canal shape, as quantified by eigenshape analysis, versus relative cochlea size.** For each semicircular canal the relationship between cochlea size and the first three eigenshape axes were investigated; the percentage of the total sample shape variance expressed by each axes was as follows: anterior ES1 28.73%, ES2 18.60% and ES3 9.32%; lateral ES1 28.29%, ES2 19.27% and ES3 11.95%; posterior ES1 31.44%, ES2 16.29% and ES3 13.19%. Anterior – top row: (i) ES1, (ii) ES2 and (iii) ES3; Lateral – middle row: (iv) ES1, (v) ES2 and (vi) ES3; Posterior – lower row: (vii) ES1, (viii) ES2 and (ix) ES3. Models represent shape change down the first three eigenshape axes, with models from high to low representing semicircular canal shape modelled at values of 0.2, 0.0 and −0.2 respectively, as shown in (i). Solid lines represent the OLS regression, Yinpterochiroptera [Old World fruit bats (orange), laryngeal echolocating Yinpterochiroptera species (blue)] and the Yangochiroptera (yellow). **B Canonical variates analysis of the (i) anterior, (ii) lateral and (iii) posterior semicircular canals utilising the shape variation represented by eigenshape axes 1**–**20, corresponding to 95% of the total sample variance.** Bat species are colour coded as follows: Old World fruit bats (orange points); echolocating Yinpterochiroptera (blue points) and Yangochiroptera (yellow points).
(TIF)

Figure S4 **The relationship between log semicircular canal size and log WTS across a sample of echolocating Yinpterochiroptera (black circles).** The measured points for *Rhinolophus philippinensis* small morph (white circle) falls beneath the 95% PI calculated for the remaining species for (A) anterior, (B) lateral and (C) posterior semicircular canals.
(TIF)

Text S1 **Supporting information.**
(DOC)
