# Supplementary figures and images for: The Evolution of Bat Vestibular Systems in the Face of Potential Antagonistic Selection Pressures for Flight and Echolocation
Source: PLoS One. 2013 Apr 24;8(4):e61998. doi: 10.1371/journal.pone.0061998 (PMC3634842; doi:10.1371/journal.pone.0061998)

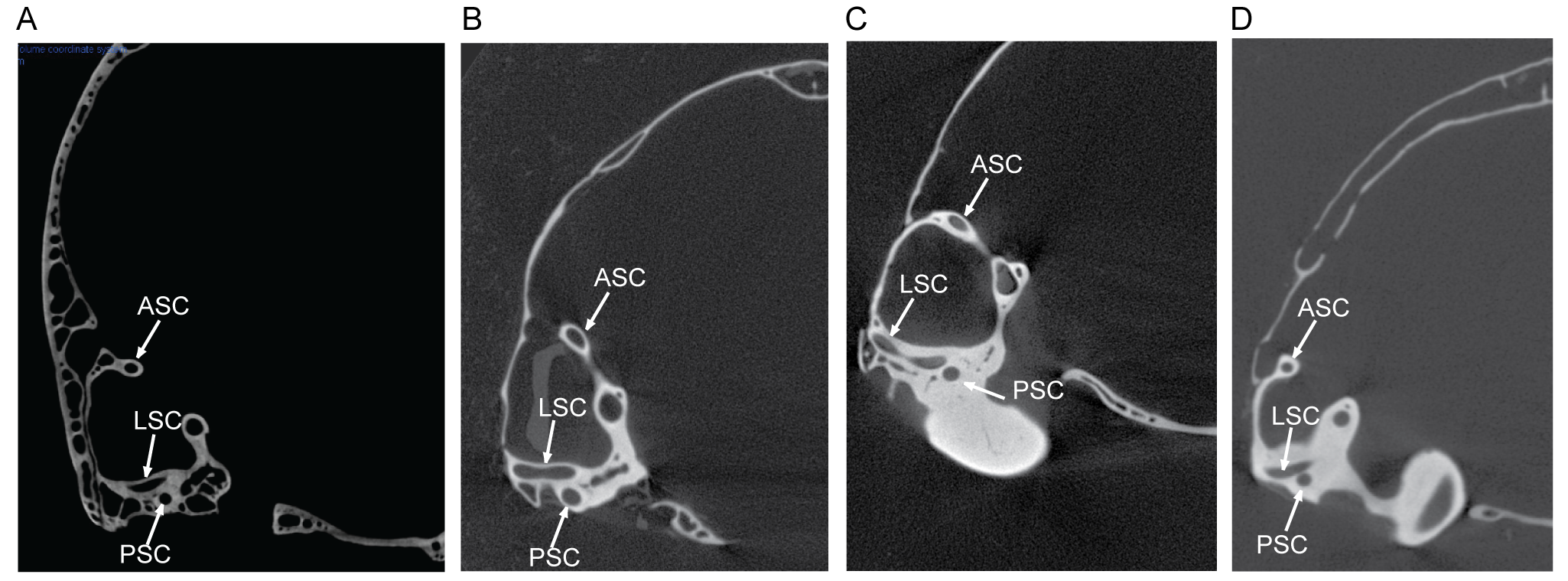

Supplement: Figure S1 — Micro-computed tomography scan slice through four bat skulls, displaying the relative position of the three semicircular canals within the skull. Scans are from the following species: (A) Pteropus rodricensis (BMNH.76.3.15.14); (B) Myotis lucifugus (BMNH.7.7.7.3359); (C) Rhinolophus ferrumequinum (58.20697) and (D) Cloeotis percivali (BMNH.66.5456). Abbreviations: ASC – anterior semicircular canal; LSC – lateral semicircular canal; PSC – posterior semicircular canal. (TIF) [file pone.0061998.s001.tif]

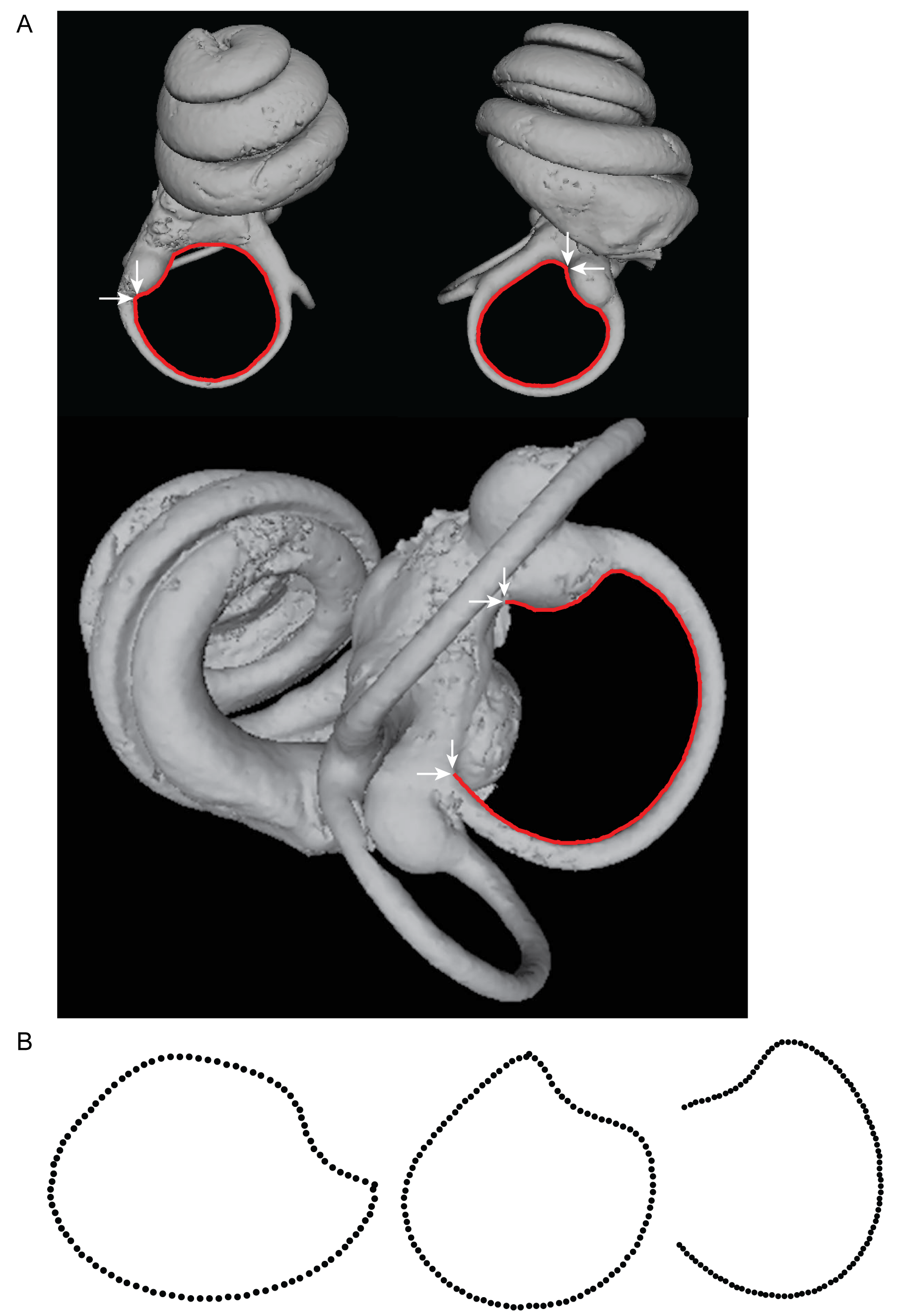

Supplement: Figure S2 — A Reconstructed inner ear volume of Trachops cirrhosus (BMNH.1924.3.1.33) indicating the orientation of each semicircular canal for outline collection. Starting points (white arrows) for each canal outline (red lines) were as follows: anterior semicircular canal (top left) - point of inflection of the ampullae; posterior semicircular canal (top right) - maximum point of curvature at apex of canal; lateral semicircular canal (bottom panel) - where canal projects freely from the base. B Mean sample shapes for (left – right) anterior, posterior and lateral semicircular canals. Outlines represent the mean semicircular canal shape of the morphological variation of 55, 54 and 58 individuals respectively, and are represented by 100 coordinate points. (TIF) [file pone.0061998.s002.tif]

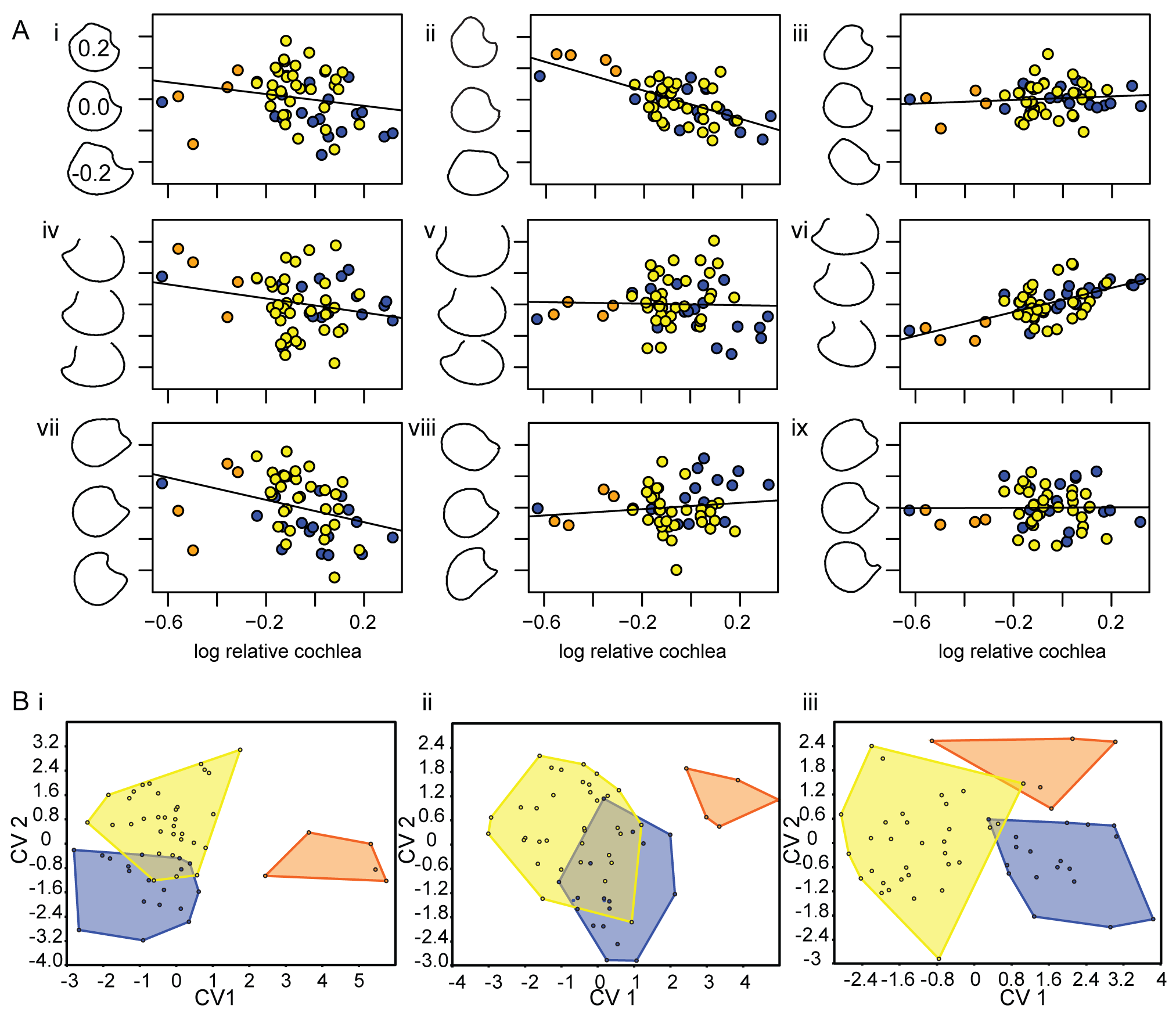

Supplement: Figure S3 — A Semicircular canal shape, as quantified by eigenshape analysis, versus relative cochlea size. For each semicircular canal the relationship between cochlea size and the first three eigenshape axes were investigated; the percentage of the total sample shape variance expressed by each axes was as follows: anterior ES1 28.73%, ES2 18.60% and ES3 9.32%; lateral ES1 28.29%, ES2 19.27% and ES3 11.95%; posterior ES1 31.44%, ES2 16.29% and ES3 13.19%. Anterior – top row: (i) ES1, (ii) ES2 and (iii) ES3; Lateral – middle row: (iv) ES1, (v) ES2 and (vi) ES3; Posterior – lower row: (vii) ES1, (viii) ES2 and (ix) ES3. Models represent shape change down the first three eigenshape axes, with models from high to low representing semicircular canal shape modelled at values of 0.2, 0.0 and −0.2 respectively, as shown in (i). Solid lines represent the OLS regression, Yinpterochiroptera [Old World fruit bats (orange), laryngeal echolocating Yinpterochiroptera species (blue)] and the Yangochiroptera (yellow). B Canonical variates analysis of the (i) anterior, (ii) lateral and (iii) posterior semicircular canals utilising the shape variation represented by eigenshape axes 1–20, corresponding to 95% of the total sample variance. Bat species are colour coded as follows: Old World fruit bats (orange points); echolocating Yinpterochiroptera (blue points) and Yangochiroptera (yellow points). (TIF) [file pone.0061998.s003.tif]

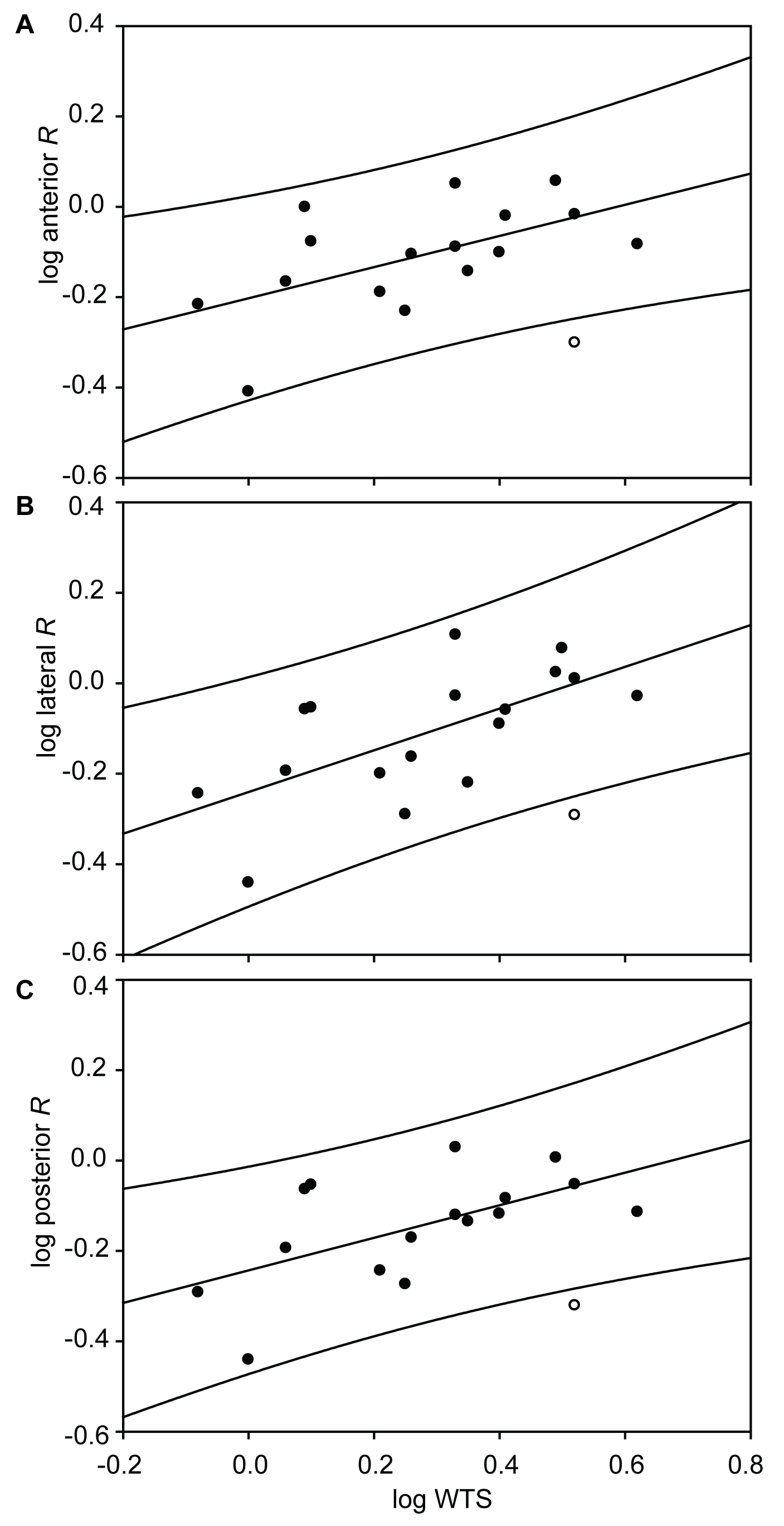

Supplement: Figure S4 — The relationship between log semicircular canal size and log WTS across a sample of echolocating Yinpterochiroptera (black circles). The measured points for Rhinolophus philippinensis small morph (white circle) falls beneath the 95% PI calculated for the remaining species for (A) anterior, (B) lateral and (C) posterior semicircular canals. (TIF) [file pone.0061998.s004.tif]
