# Supplementary material for: The Evolution of Bat Vestibular Systems in the Face of Potential Antagonistic Selection Pressures for Flight and Echolocation
Source: PLoS One. 2013 Apr 24;8(4):e61998. doi: 10.1371/journal.pone.0061998 (PMC3634842; doi:10.1371/journal.pone.0061998)
Supplement: Text S1 — Supporting information. (DOC) [file pone.0061998.s005.doc]

**SUPPORTING INFORMATION**

**The evolution of bat vestibular systems in the face of potential antagonistic selection pressures for flight and echolocation**

Kalina T.J. Davies, Paul J.J. Bates, Ibnu Maryanto, James A. Cotton, Stephen J. Rossiter

**Supplementary materials and methods**

*Study sample and acquisition of µCT-scan data*

Preservation state and method of preparation varied between the 68 specimens studied; some were complete specimens preserved in ethanol, and others prepared skulls. Specimens were all adult apart from a sub-adult *Hypsignathus monstrosus*. We were unable to include both sexes for each species, therefore sexual dimorphism and individual asymmetry in mammalian inner ears was assumed to be negligible in agreement with previous studies [1,2]. Our dataset included three documented size-morphs of *Rhinolophus philippinensis* [3]. The basal parts of all skulls were scanned, with the exception of specimen BNHM.65.3990, where the single remaining bony labyrinth was dislodged from the skull and thus scanned directly. The ‘region grower’ tool in VG Studio Max 2.0 (Volume Graphics, Heidelberg, Germany) was used to digitally dissect the low density internal voids of the bony labyrinth, i.e. the cochlea and semicircular canals, to produce a digital endocast for use in MeshLab v.1.2.2 (MeshLab Visual Computing Lab – ISTI – CNR) and Landmark v.3.0 [4]. Measurements were taken from a single labyrinth for the majority of specimens. Where multiple specimens per species were available, linear measurements were averaged prior to analysis, with the exception of the three *R. philippinensis* size morphs which might be independent taxa. Similarly where both sets of semicircular canals were available for an individual, average values were used to avoid pseudo-replication. For eigenshape analysis, when multiple specimens were available, one outline per species was chosen at random. In order to make functional interpretations based on representative individuals it was assumed that inter-specific variation is greater then intra-specific variation, which was confirmed by estimates of the variation shown by labyrinths from multiple individuals [Mean *R* ± SD, min–max (*n*): *Rhinolophus pearsonii* 0.778 ± 0.084, 0.718–0.838 (*2*); *Miniopterus schreibersii* 0.841 ± 0.02, 0.830–0.853 (*2*); *Plecotus auritus* 0.911 ± 0.05, 0.875–0.953 (*3*); *Pipistrellus pipistrellus* 0.679 ± 0.02, 0.670–0.705 (*4*); *Noctilio leporinus* 0.939 ± 0.02, 0.923–0.955 (*2*)] and both labyrinths from the same specimen [Mean *R* ± SD, min–max (n): *Rhinolophus pearsonii* 0.840 ± 0.004, 0.838–0.843 (*2*)*, Pteronotus parnellii* 0.984 ± 0.001, 0.980–0.988 (*2*); *Carollia perspicillata* 0.771 ± 0.004, 0.760–0.783 (*2*)].

*Examining allometry*

Published radius of curvature (*R*) values and associated body mass values were collected for 156 non-bat mammal species (taken from [5,6,7]), values were log_10_ transformed to normalise the variance, and log *R* was plotted against log body mass (to compare between a linear and volume metric). Here we tested whether the data followed a simple linear relationship across all taxa, or whether – due to wide variation in mammalian body size – they were better described by a discontinuous model. The relationship between these variables was explored in the R statistical package version 2.11.1 [8] using a modified protocol as outlined in Knell [9], as follows:-

1. Log-log scatterplots of the data were determined to be either: (a) a clear continuous relationship (a straight line), (b) a clear discontinuous relationship e.g. the dataset may be broken into size classes, or (c) not possible to determine the nature of the relationship.

2. For 1(b), the overlap in the X-variable (i.e. log body mass) is an important factor that needs to be taken into account as well as the position of the break-point. Relative frequency and density plots were used to verify the discontinuous relationship and, where clear, appropriate models were fitted to the data and compared using AIC.

3. For 1(c), if a simple linear regression was fitted and examination of standard diagnostic plots indicated no systematic deviation from a straight line, this was considered sufficient.

To test whether the bats measured by this study had proportionally larger semicircular canals compared to non-volant mammals, the bat *R* measures were added to the values for the 156 non-bat mammals and then the distribution examined. If a bat species’ body mass was unobtainable from field or literature sources, values from a congeneric taxon was used. A possible caveat of using body mass is that bats might be under selective constraints for small body mass [10,11]. However, the bones of bats are relatively dense compared to those of terrestrial mammals, and so contribute disproportionally to their total body mass [12].

*Geometric Morphometric analysis*

Bat semicircular canal shape was examined using eigenshape analysis [13,14]. This technique standardises overall object size and maximally aligns outlines so that only shape differences are assessed. Each 3D labyrinth volume was rotated until each approximately planar semicircular canal was maximally aligned parallel to the field of vision and then a 2D image was captured and exported for use in Image-Pro Plus v.5.1 (Media Cybernetics Inc., Bethesda, MD, USA). Outlines were captured manually, using the ‘*capture outline*’ function, along the canal outline between homologous start and end points. Anterior and posterior canal shape was captured as a closed outline and lateral canal shape as an open outline (see Fig. S2).

Points were transformed into the φ format (net angular change between adjacent points around the outline, see MacLeod [15]) to remove size and increase the efficiency of the analysis. Analysis of the curve set was then carried out using the Eigenshape2.1.exe program. This implements a singular value decomposition of the pair-wise covariance matrix of φ shape functions. The procedure summarizes the shape variation present in the sample as a series of orthogonal multivariate vectors (eigenshapes) expressing the set of variance-maximized shape trends. The lengths of these vectors (given as eigenvalues) reflect the degree to which each represents the sample variance, and the projections of the original φ shape functions onto these vectors (given as sets of eigenshape scores) represent a shape-similarity-ordination of the outlines. Models were constructed using ESModels1(2.1).exe, which allow modes of shape change to be visualised along each axes.

To summarise overall semicircular canal shape variation across bats, a canonical variates analysis (CVA) was performed on the eigenscores that explained 95% of the sample shape variance. Three groups were predefined, corresponding to the non-echolocating Old World fruit bats, the remaining (echolocating) Yinpterochiroptera and the Yangochiroptera. The CVA constructs new variables that describe the relative positions of these groups within the sample, and is used to visualise the maximum extent of separation. The CVA was performed using PAST v.2.09 [16].

*Phylogeny construction and Bayesian phylogenetic mixed models*

To control for the shared ancestry of characters, and thus test whether observed relationships were evident after accounting for phylogenetic relatedness, we used Bayesian phylogenetic mixed models (BPMMs) implemented in ‘MCMCglmm’ [17] in R v.2.11.1. These mixed-effects models seek to determine the extent to which morphological variation can be explained by fixed and random effects, in which the random effect is the covariance between taxa caused by their shared evolutionary histories. Within these models, the contribution of ‘shared evolutionary history’ is included in the model as an explanatory variable for the trait of interest, so that the effect of other traits can be analysed correctly in the presence of this non-independence. The models’ structure are identical to those of the ‘animal model’ widely used in animal breeding and quantitative genetics, a fact that can be exploited to make use of software and statistical approaches developed to fit these complex models efficiently and flexibly [18]. We compared models utilising phylogenetic information by examining the Deviance Information Criterion (DIC); with ΔDIC values ≥2 taken to indicate significantly improved model fit. For tests of fixed effects, we report the *P*_MCMC_ value, which is twice the posterior probability that a model parameter is greater or less than zero (whichever is lower), as estimated by the Markov chain, and is one possible Bayesian analogue to a two-tailed frequentist p-value.

*Running analysis* *and defining priors*

As the default settings in ‘MCMCglmm’ use improper priors which are reputed to cause numerical problems [19], in all analyses proper priors were specified. In large informative datasets, the prior should have little impact on parameter estimates, but it is still necessary to compare results from different runs to ensure the analysis is robust to prior specification [19]. Initial tests of priors suggested that estimated parameters were approximately equal; and it was decided to use a prior, with n=1, with phenotypic variance divided equally for all runs. All analyses were run with the same basic settings: animal as a random effect and with pedigree information obtained from the dated phylogeny, and with proper priors with a low degree of belief and phenotypic trait variance divided equally between genetic and residual effects. All models were run with 1,000,000 iterations with 100,000 burn in and thinning interval of 100. Autocorrelation values <0.01 were used to check for good mixing.

**Supplementary results**

*Model fitting and allometry*

From initial inspection of the distribution of published log *R* against log body mass for non-bat mammals, it was not possible to determine whether a continuous or discontinuous relationship best described the data. More importantly, fitting of a simple linear model to the data revealed that this relationship overestimated semicircular canal size at low and high body masses (Model 1 in Table S2A). Examination of standard diagnostic plots also revealed that the data were left-skewed and not strictly unimodal (data not shown). Non-linear models were not considered as there is no physiological basis to assume the relationship between semicircular canal size and body mass is non-linear.

We also assessed the suitability of two discontinuous models: Model 2, in which species were separated depending on whether they had a body mass of above or below 130g, and Model 3, in which species were split into two groups corresponding to the lateral semicircular canal data (compare the distribution of black and grey points in Fig. 3B). For Model 2 the body mass cut-off value (130g) was chosen arbitrarily after examination of the density plots and histograms (data not shown); these plots indicate a break in the data at log *R* / log body mass approximates zero, corresponding to a body mass of ~130g. Based on this body mass value of 130g, two groups were formed: one containing species with body mass <130g and one containing species with body mass >130g. Although Model 3 allows some overlap between the two clouds of points along the x-axis there is none along the y-axis, so when information from both axes are considered two discrete groups are formed. A body mass of 130 g also corresponds to the highest body mass observed in the black point group in Model 3 (see Fig. 3B). It should be noted that several members of the grey point group do have a body mass below 130 g; this could indicate that other factors, such as taxonomic constraint, affect the observed allometric relationship. Both discontinuous models appeared to fit the data well, although Model 3 (that follows the observed grouping) had a significantly better AIC score than Model 2 and was therefore used in subsequent analyses (see Table S2A). After accounting for phylogenetic relatedness, ΔDIC values again suggest that models with two different size classes fitted the data best (ΔDIC: anterior: (2) 14.080, (3) 13.895; lateral: (2) 29.998, (3) 28.149; posterior: (2) 14.820 (3) 14.778, see Table S2B). However, in models with the interaction term [Model 2 (b) and 3 (b)], the interaction itself was not significant (*P*_MCMC_: anterior 0.647; lateral 0.337; posterior 0.453). Thus while we found support for different scaling factors in large and small non-flying taxa, we did not find evidence of different allometries (i.e. slope gradients) within each group. Consequently, while there might be changes in overall magnitude of the relationship, the negative allometrical nature of relationship is of the same order.

Observed differential scaling among mammals might reflect the extreme range in body mass in this group. For example, inconsistent scaling parameters across mammalian taxa could arise from divergent selection pressures or physical constraints acting on particular groups, which will be most apparent in taxa that display extremes of body mass. Therefore it may not always be possible to fit a single simple linear model to investigate mammalian trait evolution. Bats typically display low body mass, but also show significant variation across the order [20]; therefore, when comparing the allometry of physical traits such as the vestibular system between bats and other mammals, differential scaling properties could be relevant. In fact, our analyses showed that in comparisons of the vestibular system of bats with other small-bodied mammals, a single linear regression model based on all taxa for which data is available fitted the data poorly. For this reason, we grouped taxa approximately by body mass, and calculated separate allometries. We found support for different scaling factors in large and small non-flying taxa after accounting for phylogenetic relatedness, suggesting different constraints act on these species. Thus the size categories adopted here, although perhaps imperfect, are justified and the limited overlap of size range between the two adopted classes may be due to taxonomic constraints. Similar differential scaling was previously shown in mammalian middle ear bones [21], and the lack of correlation between intraspecific semicircular canal size and body mass in some species has also led to suggestions of species-specific differential scaling properties [1]. Furthermore, the vestibular systems of cetaceans deviate markedly from the allometric relationship shown by terrestrial mammals (e.g. [5]), again highlighting that differential scaling relationships occur across mammalian groups.

**Table S1**

**A. Species and inner ear measurements used in this study.**

| Species | Species number | Semicircular canal *R* | | | Cochlea size | Source | Specimen number |
| --- | --- | --- | --- | --- | --- | --- | --- |
|  |  | anterior | lateral | posterior |  |  |  |
| *Epomophorus gambianus* | 1 | 1.29 | 1.08 | 1.28 | NA | [7] | NA |
| *Rousettus lanosus* | 2 | 1.51 | 1.13 | 1.21 | 2.52 | HZM | 16.36082 |
| *Rousettus aegyptiacus* | 3 | 1.31 | 1.07 | 1.12 | 2.28 | HZM | 107.11626 |
| *Pteropus rodricensis* | 4 | 1.40 | 1.03 | 1.17 | NA | [7] | NA |
| *Pteropus rodricensis* | NA | NA | NA | NA | NA | NHM | BMNH.76.3.15.14 |
| *Hypsignathus monstrosus* | 5 | 1.48 | 1.26 | 1.20 | 2.39 | HZM | 1.3518 |
| *Pteropus sp.* | 6 | 1.79 | 1.54 | 1.58 | 2.92 | QMUL | NA |
| *Pteropus giganteus* | 7 | 1.53 | 1.31 | 1.33 | 3.10 | [5,6] | NA |
| *Rhinolophus cornutus* | 8 | 0.65 | 0.63 | 0.57 | NA | [6] | NA |
| *Rhinolophus philippinensis(S)* | 9 | 0.50 | 0.51 | 0.48 | 1.99 | MZB | MZB22913 |
| *Rhinolophus philippinensis(M)* | 10 | 0.96 | 1.02 | 0.89 | 3.67 | MZB | MZB22897 |
| *Rhinolophus philippinensis(L)* | NA | NA | 1.20 | NA | 4.47 | MZB | MZB22910 |
| *Rhinolophus pearsonii_1* | 11 | 0.84 | 0.94 | 0.79 | 3.05 | HZM | 14.3511 |
| *Rhinolophus pearsonii_2* | NA | 0.81 | NA | 0.75 | 3.41 | HZM | NA |
| *Rhinolophus megaphyllus* | 12 | 1.13 | 1.28 | 1.07 | 4.44 | NHM | BMNH.1903.8.3.3 |
| *Rhinolophus marshallii* | 13 | 0.82 | 0.94 | 0.76 | 3.41 | HZM | NA |
| *Rhinolophus affinis* | 14 | 0.84 | 0.88 | 0.88 | 2.99 | HZM | 29.35223 |
| *Rhinolophus ferrumequinum* | 15 | 0.79 | 0.81 | 0.76 | 2.95 | HZM | 58.20697 |
| Cloeotis percivali | 16 | 0.39 | 0.36 | 0.36 | 1.81 | HZM | 13.4765 |
| Cloeotis percivali | NA | NA | NA | NA | NA | NHM | BMNH.66.5456 |
| *Hipposideros fulvus* | 17 | 0.59 | 0.51 | 0.53 | 1.94 | HZM | 3.28778 |
| *Hipposideros ridleyi* | 18 | 0.72 | 0.60 | 0.73 | 2.38 | NHM | BMNH.1983.423 |
| *Hipposideros gigas* | 19 | 1.14 | 1.06 | 1.02 | 3.72 | HZM | 3.1164 |
| *Rhinopoma hardwickii* | 20 | 0.96 | 0.87 | 0.83 | 2.24 | HZM | 37.9152 |
| *Rhinopoma microphyllum* | 21 | 1.00 | 0.88 | 0.86 | 2.19 | NHM | BMNH.1968.453 |
| *Lavia frons* | 22 | 1.01 | 0.86 | 0.86 | 2.19 | HZM | 30.25025 |
| *Megaderma spasma* | 23 | 0.79 | 0.69 | 0.68 | 1.74 | NHM | BMNH.1912.11.28.32 |
| *Cardioderma cor* | 24 | 1.54 | 1.29 | 1.24 | 2.98 | NHM | BMNH.1975.2453 |
| *Macroderma gigas* | 25 | 0.61 | 0.57 | 0.51 | 1.18 | NHM | BMNH.1892.5.20.2 |
| *Craseonycteris thonglongyai* | 26 | 0.68 | 0.64 | 0.64 | 1.88 | HZM | 1.34982 |
| *Miniopterus schreibersii* | 27 | 0.83 | 0.75 | 0.70 | 1.86 | NHM | BMNH.62.1443 |
| *Miniopterus schreibersii* | 27 | 0.85 | 0.71 | 0.77 | 1.91 | HZM | 247.22505 |
| *Murina tubinaris* | 28 | 0.92 | 0.64 | 0.62 | 2.18 | HZM | NA |
| *Murina suilla* | 29 | 0.75 | 0.65 | 0.62 | 1.89 | NHM | BMNH.1879.11.15.16 |
| *Myotis muricola* | 30 | 0.74 | 0.58 | 0.58 | 2.11 | HZM | NA |
| *Myotis macrodactylus* | 31 | 0.74 | 0.56 | 0.57 | NA | [6] | NA |
| *Pipistrellus pipistrellus_1* | 32 | 0.67 | 0.56 | 0.54 | 1.60 | QMUL | NA |
| *Pipistrellus pipistrellus_2* | 32 | 0.68 | 0.58 | 0.58 | 1.59 | QMUL | NA |
| *Pipistrellus pipistrellus_3* | 32 | 0.67 | 0.57 | 0.55 | 1.58 | QMUL | NA |
| *Pipistrellus pipistrellus_4* | 32 | 0.71 | 0.59 | 0.58 | 1.69 | QMUL | NA |
| *Pipistrellus pipistrellus* | 33 | 0.88 | 0.59 | 0.63 | NA | [6] | NA |
| *Myotis lucifugus* | 34 | 0.71 | 0.57 | 0.58 | 1.54 | NHM | BMNH.7.7.7.3359 |
| *Myotis lucifugus* | 35 | 0.88 | 0.69 | 0.67 | NA | [6] | NA |
| *Plecotus auritus* | 36 | 0.95 | 0.68 | 0.78 | 1.81 | QMUL | NA |
| *Plecotus auritus* | 36 | 0.88 | 0.64 | 0.71 | 1.75 | QMUL | NA |
| *Plecotus auritus* | 36 | 0.91 | 0.70 | 0.71 | 1.80 | QMUL | NA |
| *Lasiurus borealis* | 37 | 0.76 | 0.66 | 0.65 | 1.90 | HZM | 4.3341 |
| *Eptesicus fuscus* | 38 | 0.87 | 0.77 | 0.67 | NA | [6] | NA |
| *Scotomanes ornatus* | 39 | 0.94 | 0.77 | 0.78 | 2.12 | HZM | NA |
| *Scotophilus* *kuhlii* | 40 | 0.90 | 0.85 | 0.69 | 2.17 | HZM | NA |
| *Nyctalus lasiopterus aviator* | 41 | 1.01 | 0.99 | 0.85 | NA | [6] | NA |
| *Scotophilus robustus* | 42 | 1.06 | 0.76 | 0.79 | NA | [7] | NA |
| *Murina cyclotis* | NA | NA | 0.63 | 0.67 | 1.82 | NHM | BMNH.16.3.25.29 |
| Noctilio leporinus | 43 | 0.92 | 0.83 | 0.76 | 2.70 | NHM | BMNH.1928.7.21.35 |
| Noctilio leporinus | 43 | 0.96 | 0.91 | 0.78 | 2.97 | HZM | 12.15977 |
| *Thyroptera sp.* | 44 | 0.69 | 0.65 | 0.54 | 2.19 | QMUL | NA |
| Pteronotus *(Chilonycteris) macleayii grisea* | 45 | 0.79 | 0.64 | 0.63 | 1.92 | NHM | BMNH.65.3990 |
| Pteronotus davyi | 46 | 0.69 | 0.61 | 0.60 | 1.85 | HZM | 8.16031 |
| Pteronotus *parnellii* | 47 | 0.99 | 0.91 | 0.79 | 3.05 | HZM | 5.21236 |
| Mormoops megaphylla | 48 | 0.91 | 0.71 | 0.70 | 1.93 | HZM | 2.1603 |
| Anoura geoffroyi | 49 | 0.65 | 0.55 | 0.58 | 1.40 | NHM | BMNH.1914.5.21.4 |
| Carollia perspicillata | 50 | 0.77 | 0.65 | 0.68 | 1.84 | HZM | 140.29127 |
| Centurio senex | 51 | 0.75 | 0.61 | 0.64 | 2.04 | HZM | 1.13198 |
| Desmodus rotundus | 52 | 1.15 | 0.84 | 1.01 | 2.21 | HZM | 41.11631 |
| Tonatia silvicola | 53 | 0.92 | 0.80 | 0.79 | 2.39 | NHM | BMNH.1954.322 |
| Trachops cirrhosus | 54 | 1.05 | 0.91 | 0.87 | 2.49 | NHM | BMNH.1924.3.1.33 |
| Artibeus jamaicensis | 55 | 0.91 | 0.85 | 0.77 | 2.42 | NHM | BMNH.1907.1.1.684 |
| *Molossus molossus* | 56 | 0.77 | 0.62 | 0.66 | 2.31 | HZM | 61.28841 |
| *Molossus molossus* | 57 | 0.95 | 0.49 | 0.72 | NA | [7] | NA |
| Cheiromeles torquatus | 58 | 2.00 | 1.74 | 1.70 | 4.86 | NHM | BMNH.1844.10.17.7 |
| *Tadarida brasiliensis* | NA | 0.75 | 0.69 | NA | 1.76 | NHM | BMNH.1960.482 |
| *Nycteris thebaica* | 59 | 0.80 | 0.70 | 0.64 | 1.90 | HZM | 214.35929 |
| *Natalus stramineus saturates* | 60 | 0.75 | 64.00 | 0.53 | 1.74 | HZM | 8.7055 |
| *Rhynchonycteris naso* | 61 | 0.61 | 0.53 | 0.53 | 1.74 | HZM | 34.586 |
| *Peropteryx macrotis* | 62 | 0.73 | 0.68 | 0.65 | 1.96 | HZM | 3.18512 |
| *Saccopteryx bilineata* | 63 | 0.71 | 0.63 | 0.59 | 2.14 | HZM | 8.16002 |
| *Taphozous melanopogon* | 64 | 1.00 | 0.90 | 0.85 | 2.28 | HZM | 18.30235 |
| *Taphozous peli* | 65 | 1.21 | 1.07 | 1.08 | 3.01 | HZM | 2.1845 |

Species code refers to species number in Fig. 4.

**B. Body mass and wing morphology parameters and sources used in this study.**

| Family | Species | Body mass (g) | ref: | WAR | ref: | WL | ref: | WTS | ref: | Comments: |
| --- | --- | --- | --- | --- | --- | --- | --- | --- | --- | --- |
| Pteropodidae | *Pteropus rodricensis* | 338 | a | 6.3 | a | 46.6 | a | 1.01 | a |  |
|  | *Pteropus sp.* | 1175* | [6] | 5.3* | [22] | 20.3* | [22] | 1.24** | [20] | **P. giganteus*,  ***P. vampyrus* |
|  | *Pteropus giganteus* | 1175 | [6] | 5.3 | [22] | 20.3 | [22] | 1.24* | [20] | **P. vampyrus* |
|  | *Rousettus lanosus* | 140 | [20] | 5.9* | [20] | 24.6* | [20] | 1.45* | [20] | **R. aegyptiacus* |
|  | *Rousettus aegyptiacus* | 140 | [20] | 5.9 | [20] | 24.6 | [20] | 1.45 | [20] |  |
|  | *Hypsignathus monstrosus* | 427 | [20] | 6.7 | [20] | 36.3 | [20] | 1.76 | [20] |  |
|  | *Epomophorus gambianus* | 158 | [20] | 5.9 | [20] | 27.0 | [20] | NA | - |  |
| Rhinolophidae | *Rhinolophus philippinensis (S)* | 6.5 | b | 6.7 | c | 4.6 | c | 3.34 | c |  |
|  | *Rhinolophus philippinensis (M)* | 7 | b | 6.7 | c | 4.6 | c | 3.34 | c |  |
|  | *Rhinolophus philippinensis (L)* | 12 | b | 9.0 | c | 7.9 | c | 2.53 | c |  |
|  | *Rhinolophus ferrumequinum* | 22.6 | [20] | 6.1 | [20] | 12.2 | [20] | 2.13 | [20] |  |
|  | *Rhinolophus megaphyllus* | 9.8 | [20] | 6.1 | [20] | 7.4 | [20] | 2.15 | [20] |  |
|  | *Rhinolophus pearsonii* | 8.8 | d | 4.4 | e | 9.4 | e | 4.17 | e |  |
|  | *Rhinolophus marshalli* | 6.4 | [23] | 6.4 | e | 12.4 | e | 3.16* | [24] | * *R. macrotis* |
|  | *Rhinolophus affinis* | 12.6 | d | 8.1 | [25] | 8.6 | f | 1.27 | f |  |
|  | *Rhinolophus cornutus* | 6.1 | [6] | 5.2 | [26] | 13.9 | e | 1.63 | e |  |
| Hipposideridae | *Hipposideros ridleyi* | 9.3 | [27] | 6.0 | [27] | 6.1 | [27] | 2.25 | [27] |  |
|  | *Hipposideros fulvus* | 8.5 | [28] | 5.5 | e | 14.1 | e | 1.80 | e |  |
|  | *Hipposideros gigas* | 89 | [20] | 7.7* | [20] | 15.7* | [20] | 3.1* | [29] | **H. commersoni* |
|  | *Cloeotis percivali* | 4.0 | [30] | 4.8 | e | 11.2 | e | 1.01 | e |  |
| Rhinopomatidae | *Rhinopoma microphyllum* | 32 | [20] | 8.0 | [20] | 20.5 | [20] | 1.24 | [20] |  |
|  | *Rhinopoma hardwickii* | 16.3 | [20] | 6.9 | [20] | 14.0 | [20] | 2.56 | [20] |  |
| Megadermatidae | *Macroderma gigas* | 123 | [20] | 6.1 | [20] | 16.8 | [20] | 0.83 | [20] |  |
|  | *Cardioderma cor* | 28 | [31] | 5.7 | [31] | 9.6 | [20] | 1.7 | [31] |  |
|  | *Megaderma spasma* | 27 | [27] | 5.0 | [20] | 7.8 | [27] | 1.83 | [20] |  |
|  | *Lavia frons* | 34.8 | d | 5.4 | [20] | 12.0 | [20] | 1.69 | [20] |  |
| Craseonycteridae | *Craseonycteris thonglongyai* | 2.5 | d | 7.1 | [20] | 5.2 | [20] | 1.14 | [20] |  |
| Miniopteridae | *Miniopterus schreibersii* | 14.2 | [20] | 7.0 | [20] | 10.2 | [20] | 1.03 | [20] |  |
| Vespertilionidae | *Murina tubinaris* | 4.75 | [32] | 5.2 | e | 7.0 | e | 0.62 | e |  |
|  | *Murina suilla* | 3.9 | [27] | 6.0 | [27] | 5.1 | [27] | 1.607 | [27] |  |
|  | *Murina cyclotis* | 11.3 | [27] | 6.0 | [27] | 7.5 | [27] | 1.337 | [27] |  |
|  | *Myotis lucifugus* | 8.1 | [6] | 6.0 | [20] | 7.5 | [20] | 3.2 | [20] |  |
|  | *Myotis muricola* | 4.85 | [33] | 4.8 | e | 6.3 | e | 2.26* | [20] | **M. mystacinus* |
|  | *Myotis macrodactylus* | 6.5 | [6] | 6.3* | [20] | 7* | [20] | 2.05* | [20] | **M. daubentonii* |
|  | *Scotophilus kuhlii* | 34.5* | [20] | 5.8 | e | 15.0* | [20] | NA | - | **S. heathii* |
|  | *Scotophilus robustus* | 83 | [7] | 6.9 | e | NA | - | NA | - |  |
|  | *Scotomanes ornatus* | 21.8 | [34] | 5.9 | e | 21.8 | e | NA | - |  |
|  | *Lasiurus borealis* | 16.7 | [20] | 6.7 | [20] | 14.0 | [20] | 1.26 | [20] |  |
|  | *Plecotus auritus* | 9 | [20] | 5.7 | [20] | 7.1 | [20] | 1.43 | [20] |  |
|  | *Pipistrellus pipistrellus* | 6.55 | [6] | 7.5 | [20] | 8.1 | [20] | 1.74 | [20] |  |
|  | *Eptesicus fuscus* | 18.79 | [6] | 6.4 | [20] | 9.4 | [20] | 1.09 | [20] |  |
|  | *Nyctalus lasiopterus aviator* | 34.8 | [6] | 7.2 | [35] | 19.7 | [35] | 0.99* | [36] | **N. noctula* |
| Noctilionidae | *Noctilio leporinus* | 59 | [20] | 9.0 | [20] | 15.2 | [20] | 2.23 | [20] |  |
| Thyropteridae | *Thyroptera sp.* | 3.1* | [20] | 5.9* | [20] | 4.1* | [20] | 1.75* | [20] | **T. discifera* |
| Mormoopidae | *Pteronotus macleayi grisea* | 5.35 | [37] | 7.6 | [37] | 4.6 | [37] | NA | - |  |
|  | *Pteronotus parnellii* | 14.7 | [38] | 6.7 | [38] | 7.9 | [38] | 1.54 | [20] |  |
|  | *Pteronotus davyi* | 10.9 | [20] | 8.3 | [20] | 8.0 | [20] | 1.36 | [20] |  |
|  | *Mormoops megaphylla* | 17 | [20] | 7.1 | [20] | 11.2 | [20] | 1.1 | [20] |  |
| Phyllostomidae | *Artibeus jamaicensis* | 47 | [20] | 6.4 | [20] | 16.6 | [20] | 1.27* | [20] | **A. lituratus* |
|  | *Tonatia silvicola* | 32.85 | [39] | 5.0 | [40] | 15.7 | [40] | NA | - |  |
|  | *Trachops cirrhosus* | 43.8 | [20] | 6.3 | [20] | 15.3 | [20] | NA | - |  |
|  | *Desmodus rotundus* | 28.5 | [20] | 6.7 | [20] | 14.0 | [20] | 1.38 | [20] |  |
|  | *Anoura geoffroyi* | 14.1 | [20] | 7.2 | [20] | 12.5 | [20] | 3 | [20] |  |
|  | *Carollia perspicillata perspicillata* | 19.1 | [20] | 6.1 | [20] | 11.4 | [20] | 2.22 | [20] |  |
|  | *Centurio senex* | 22 | [20] | NA | - | NA | - | 0.93 | [20] |  |
| Molossidae | *Cheiromeles torquatus* | 135.5 | [20] | 8.6* | [20] | 35.7 | [20] | NA | - | **Cheiromeles sp* |
|  | *Molossus molossus* | 16.2 | [20] | 8.7 | [20] | 16.0 | [20] | NA | - |  |
|  | *Tadarida brasiliensis* | 12.5 | [20] | 8.2 | [20] | 11.5 | [20] | 1.48 | [20] |  |
| Nycteridae | *Nycteris thebaica* | 11 | [20] | 5.5 | [20] | 6.3 | [20] | 4.5 | [29] |  |
| Natalidae | *Natalus stramineus saturates* | 3.9 | [20] | 5.8 | [20] | 3.9 | [20] | 2.83 | [20] |  |
| Emballonuridae | *Taphozous peli* | 95 | [22] | 10.2* | e | 69.5* | e | NA | - | * *T. saccolaimus* |
|  | *Taphozous melanopogon* | 24 | d | 10.0 | [20] | 25.9 | [20] | 1.1* | [29] | * *T. mauritanus* |
|  | *Peropteryx macrotis* | 4.4 | [41] | 6.8 | e | 10.1 | e | 0.78 | e |  |
|  | *Saccopteryx bilineata* | 7.5 | [20] | 6.1 | [20] | 5.9 | [20] | 1.53 | [20] |  |
|  | *Rhynchonycteris naso* | 3.9 | [20] | 6.5 | [20] | 4.3 | [20] | NA | - |  |

**Additional sources:** (a) A Walsh, Lubee Bat Conservancy, (b) Recorded by SJ Rossiter and T Kingston, (c) Calculated from wing traces, (d) Recorded by HZM, (e) Calculated from specimen, (f) MJ Struebig (Unpublished data).

Abbreviations: *R* – Semicircular canal radius of curvature; NHM – Natural History Museum London; QMUL – Queen Mary University of London; HZM – Harrison Zoological Museum; MZB – Museum Zoological Bogor; NA – data missing; WAR – wing aspect ratio; WL – wing loading; WTS – wing tip shape. *denotes substitute species.

**Table S2**

**A. OLS and RMA model comparison of the relationship between semicircular canal size and body mass in non-volant mammals*.**

| Model tested: | Parameter | ASCC | LSCC | PSCC |
| --- | --- | --- | --- | --- |
| 1 log *R*  = A log BM^0.33^ + C | A *OLS (RMA)* | 0.42 (0.49) | 0.44 (0.51) | 0.46 (0.51) |
|  | C *OLS (RMA)* | -0.16 (-0.23) | -0.27 (-0.34) | -0.24 (-0.30) |
|  | *r* | 0.86 | 0.86 | 0.90 |
|  | *R*^2^(Adj.) | 0.74 (0.74) | 0.74 (0.74) | 0.80 (0.80) |
|  | AIC | -333.30 | -323.94 | -366.85 |
| 2 (a) log *R* = log BM^0.33^ + size class(i) | a_1_ | 0.33 | 0.36 | 0.38 |
|  | c_1_ | -0.15 | -0.26 | -0.24 |
|  | a_2_ | 0.33 | 0.36 | 0.38 |
|  | c_2_ | -0.05 | -0.16 | -0.15 |
|  | *R*^2^(Adj.) | 0.77 (0.77) | 0.77 (0.77) | 0.82 (0.82) |
|  | AIC | -351.52 | -338.37 | -380.58 |
| 2 (b) log *R* = log BM^0.33^ * size class(i) | a_1_ | 0.58 | 0.78 | 0.62 |
|  | c_1_ | -0.29 | -0.43 | -0.37 |
|  | a_2_ | 0.31 | 0.32 | 0.36 |
|  | c_2_ | -0.02 | -0.13 | -0.13 |
|  | *R*^2^(Adj.) | 0.78 (0.78) | 0.79 (0.79) | 0.83 (0.83) |
|  | AIC | -356.18 | -354.28 | -385.73 |
| 3 (a) log *R* = log BM^0.33^ + size class(ii) | a_1_ | 0.34 | 0.34 | 0.38 |
|  | c_1_ | -0.20 | -0.32 | -0.28 |
|  | a_2_ | 0.34 | 0.34 | 0.38 |
|  | c_2_ | -0.06 | -0.14 | -0.15 |
|  | *R*^2^(Adj.) | 0.79 (0.79) | 0.81 (0.81) | 0.85 (0.84) |
|  | AIC | -364.63 | -369.96 | -402.45 |
| 3 (b) log *R* = log BM^0.33^ * size class(ii) | a_1_ | 0.39 | 0.52 | 0.39 |
|  | c_1_ | -0.22 | -0.40 | -0.29 |
|  | a_2_ | 0.34 | 0.33 | 0.37 |
|  | c_2_ | -0.05 | -0.14 | -0.15 |
|  | *R*^2^(Adj.) | 0.79 (0.78) | 0.82 (0.81) | 0.84 (0.84) |
|  | AIC | -362.81 | -370.29 | -400.47 |

**B**. **Model comparisons non-volant mammals taking into account the phylogenetic relatedness of species.**

| Model tested: | Parameter | ASCC | LSCC | PSCC |
| --- | --- | --- | --- | --- |
| 1 log *R*  = A log BM^0.33^ + C | log BM^0.33^ | 0.437 | 0.425 | 0.443 |
|  | intercept | -0.191 | -0.278 | -0.248 |
|  | *P_MCMC_*(log BM^0.33^) | <1x 10^-4^ | <1x 10^-4^ | <1x 10^-4^ |
|  | *P_MCMC_*(intercept) | <1x 10^-4^ | <1x 10^-4^ | <1x 10^-4^ |
|  | DIC | -509.40 | -503.26 | -532.81 |
| 3 (a) log *R* = log BM^0.33^ + size class(ii) | log BM^0.33^ | 0.392 | 0.361 | 0.401 |
|  | small size | -0.090 | -0.126 | -0.084 |
|  | intercept | -0.129 | -0.190 | –0.190 |
|  | *P_MCMC_*(log BM^0.33^) | <1x 10^-4^ | <1x 10^-4^ | <1x 10^-4^ |
|  | *P_MCMC_*(small size) | <1x 10^-4^ | <1x 10^-4^ | <1x 10^-4^ |
|  | *P_MCMC_*(intercept) | 0.003 | <1x 10^-4^ | <1x 10^-4^ |
|  | DIC | -523.48 | -533.26 | -547.63 |
| 3 (b) log *R* = log BM^0.33^ * size class(ii) | log BM^0.33^ | 0.394 | 0.357 | 0.404 |
|  | small size | -0.060 | -0.185 | -0.040 |
|  | intercept | -0.130 | -0.185 | -0.194 |
|  | interaction | -0.062 | 0.123 | -0.093 |
|  | *P_MCMC_*(log BM^0.33^) | <1x 10^-4^ | <1x 10^-4^ | <1x 10^-4^ |
|  | *P_MCMC_*(small size) | 0.372 | 0.004 | 0.528 |
|  | *P_MCMC_*(intercept) | 0.003 | <1x 10^-4^ | <1x 10^-4^ |
|  | *P_MCMC_*(interaction) | 0.647 | 0.337 | 0.453 |
|  | DIC | -523.30 | -531.41 | -547.59 |

**C. Model comparisons including non-volant mammals and bats taking into account the phylogenetic relatedness of species.**

| Model tested: | Parameter | ASCC | LSCC | PSCC |
| --- | --- | --- | --- | --- |
| 1 log *R*  = A log BM^0.33^ + C | log BM^0.33^ | 0.454 | 0.436 | 0.460 |
|  | intercept | -0.210 | -0.287 | -0.269 |
|  | *P_MCMC_*(log BM^0.33^) | <1 x 10^-4^ | <1 x 10^-4^ | <1 x 10^-4^ |
|  | *P_MCMC_*(intercept) | <1 x 10^-4^ | <1 x 10^-4^ | <1 x 10^-4^ |
|  | DIC | -606.57 | -592.27 | -624.74 |
| 3 (a) log *R* = log BM^0.33^ + size class(ii) | log BM^0.33^ | 0.408 | 0.381 | 0.419 |
|  | small size | -0.073 | -0.086 | -0.066 |
|  | intercept | -0.148 | -0.214 | -0.213 |
|  | *P_MCMC_*(log BM^0.33^) | <1 x 10^-4^ | <1 x 10^-4^ | <1 x 10^-4^ |
|  | *P_MCMC_*(small size) | 0.001 | <1 x 10^-4^ | 0.001 |
|  | *P_MCMC_*(intercept) | <5 x 10^-4^ | <1 x 10^-4^ | <1 x 10^-4^ |
|  | DIC | -613.88 | -605.02 | -628.68 |
| 3 (b) log *R* = log BM^0.33^ * size class(ii) | log BM^0.33^ | 0.401 | 0.366 | 0.413 |
|  | small size | -0.098 | -0.141 | -0.087 |
|  | intercept | -0.142 | -0.197 | -0.208 |
|  | interaction | 0.051 | 0.109 | 0.043 |
|  | *P_MCMC_*(log BM^0.33^) | <1 x 10^-4^ | <1 x 10^-4^ | <1 x 10^-4^ |
|  | *P_MCMC_*(small size) | 0.016 | 0.001 | 0.025 |
|  | *P_MCMC_*(intercept) | 0.001 | <1 x 10^-4^ | <1 x 10^-4^ |
|  | *P_MCMC_*(interaction) | 0.450 | 0.130 | 0.515 |
|  | DIC | -613.11 | -607.59 | -627.95 |
| 3 (a) log *R* = log BM^0.33^ + size class(ii) | log BM^0.33^ | 0.406 | 0.384 | 0.415 |
| + Flight | small size | -0.072 | -0.089 | -0.063 |
|  | flight | 0.013 | -0.027 | 0.035 |
|  | intercept | -0.159 | -0.191 | -0.241 |
|  | *P_MCMC_*(log BM^0.33^) | <1 x 10^-4^ | <1 x 10^-4^ | <1 x 10^-4^ |
|  | *P_MCMC_*(small size) | 0.001 | <1 x 10^-4^ | 0.002 |
|  | *P_MCMC_*(flight) | 0.745 | 0.536 | 0.391 |
|  | *P_MCMC_*(intercept) | 0.004 | 0.001 | <1 x 10^-4^ |
|  | DIC | -613.69 | -605.14 | -628.41 |
| 3 (a) log *R* = log BM^0.33^ + size class(ii) | log BM^0.33^ | 0.407 | 0.385 | 0.417 |
| + Echolocation | small size | -0.070 | -0.099 | -0.061 |
|  | echolocation | 0.009 | -0.052 | 0.022 |
|  | intercept | -0.157 | -0.167 | -0.232 |
|  | *P_MCMC_*(log BM^0.33^) | <1 x 10^-4^ | <1 x 10^-4^ | <1 x 10^-4^ |
|  | *P_MCMC_*(small size) | 0.004 | <5 x 10^-4^ | 0.008 |
|  | *P_MCMC_*(echolocation) | 0.798 | 0.173 | 0.525 |
|  | *P_MCMC_*(intercept) | 0.002 | 0.003 | <1 x 10^-4^ |
|  | DIC | -613.24 | -605.11 | -628.20 |

* Size class (i) body mass cut-off of 130g and size class (ii) taxa divided into large and small groups according to data.

Abbreviations: *R* – Semicircular canal radius of curvature; A – anterior; L – lateral, P – posterior; SCC – semicircular canal; BM – body mass; a – regression coefficient; c – intercept; OLS – ordinary least squares; RMA – reduced major axis; AIC – Akaike Information Criterion; *P_MCMC_* – probability; DIC – Deviance Information Criterion.

**Table S3**

**Model comparison of the relationship between log semicircular canal *R* and log relative cochlea size, with CF echolocation as an additional factor and taking into account the phylogenetic relatedness of species.**

| Model tested | Parameter | ASCC | LSCC | PSCC |
| --- | --- | --- | --- | --- |
| log *R* = log relative cochlea | log relative cochlea | 0.817 | 0.844 | 0.792 |
|  | intercept | -0.377 | -0.476 | -0.446 |
|  | *P_MCMC_*(log relative cochlea) | <1 x 10^-4^ | <1 x 10^-4^ | <1 x 10^-4^ |
|  | *P_MCMC_* (intercept) | <1 x 10^-4^ | <1 x 10^-4^ | <1 x 10^-4^ |
|  | DIC | -248.37 | -250.17 | -225.22 |
| log *R* = log relative cochlea + CF | log relative cochlea | 0.865 | 0.879 | 0.832 |
|  | CF | 0.155 | 0.105 | 0.122 |
|  | intercept | -0.501 | -0.558 | -0.541 |
|  | *P_MCMC_* (log relative cochlea) | <1 x 10^-4^ | <1 x 10^-4^ | <1 x 10^-4^ |
|  | *P_MCMC_* (CF) | <5 x 10^-4^ | 0.010 | 0.003 |
|  | *P_MCMC_* (intercept) | <1 x 10^-4^ | <1 x 10^-4^ | <1 x 10^-4^ |
|  | DIC | -250.86 | -250.74 | -228.48 |

Abbreviations: *R* – Semicircular canal radius of curvature; CF – constant frequency echolocation; *P_MCMC_* – probability; DIC – Deviance Information Criterion.

**Table S4**

**A. Model comparisons of the relationship between semicircular canal shape and log relative cochlea** **size.**

| Model tested: | Parameter | ASCC | LSCC | PSCC |
| --- | --- | --- | --- | --- |
| ES1 = A log relative cochlea + C | A | -0.094 | -0.112 | -0.178 |
|  | C | -0.002 | -0.003 | -0.011 |
|  | SE (DF) | 0.084 (53) | 0.087 (55) | 0.091 (51) |
|  | *r* | -0.20 | -0.24 | -0.33 |
|  | T test | -1.50 | -1.80 | -2.52 |
|  | *R*^2^ (Adj.) | 0.04 (0.02) | 0.06 (0.04) | 0.11 (0.09) |
|  | F-statistic | 2.26 | 3.23 | 6.36 |
|  | *P* | 0.14 | 0.08 | 0.01 |
| ES2 = A log relative cochlea + C | A | -0.231 | -0.01 | 0.05 |
|  | C | -0.017 | 0.00 | 0.01 |
|  | SE (DF) | 0.052 (53) | 0.077 (55) | 0.068 (51) |
|  | *r* | -0.63 | -0.03 | 0.13 |
|  | T test | -5.66 | -0.22 | 0.95 |
|  | *R*^2^ (Adj.) | 0.40 (0.39) | 0.00 (-0.02) | 0.02 (0.00) |
|  | F-statistic | 34.88 | 0.05 | 0.90 |
|  | *P* | 2.55 x 10^-7^ | 0.83 | 0.35 |
| ES3 = A log relative cochlea + C | A | 0.029 | 0.192 | 0.00 |
|  | C | 0.003 | 0.014 | 0.00 |
|  | SE (DF) | 0.048 (53) | 0.045 (55) | 0.063 (51) |
|  | *r* | 0.11 | 0.63 | 0.01 |
|  | T test | 0.81 | 5.95 | 0.06 |
|  | *R*^2^ (Adj.) | 0.01 (-0.01) | 0.39 (0.38) | 0.00 (-0.02) |
|  | F-statistic | 0.65 | 35.41 | 0.00 |
|  | *P* | 0.42 | 1.93 x 10^-7^ | 0.95 |

**B. Model comparisons of the relationship between semicircular canal shape, body mass and cochlea size taking into account the phylogenetic relatedness of species.**

| Model tested: |  | ASCC | LSCC | PSCC |
| --- | --- | --- | --- | --- |
| ES1 = log BM^0.33^ | log BM^0.33^ | 0.056 | 0.140 | 0.081 |
|  | intercept | -0.031 | -0.032 | -0.044 |
|  | *P_MCMC_*(log BM^0.33^) | 0.381 | 0.058 | 0.291 |
|  | *P_MCMC_*(intercept) | 0.412 | 0.460 | 0.315 |
|  | DIC | -151.60 | -143.55 | -115.42 |
| ES1 = log BM^0.33^ + log cochlea | log BM^0.33^ | 0.016 | 0.094 | 0.137 |
|  | log cochlea | 0.117 | 0.142 | -0.180 |
|  | intercept | -0.054 | -0.061 | -0.006 |
|  | *P_MCMC_*(log BM^0.33^) | 0.819 | 0.260 | 0.111 |
|  | *P_MCMC_*(log cochlea) | 0.249 | 0.196 | 0.160 |
|  | *P_MCMC_*(intercept) | 0.210 | 0.220 | 0.914 |
|  | DIC | -160.27 | -143.76 | -112.59 |
| ES2 = log BM^0.33^ | log BM^0.33^ | 0.111 | -0.156 | 0.077 |
|  | intercept | -0.047 | 0.070 | -0.028 |
|  | *P_MCMC_*(log BM^0.33^) | 0.033 | 0.012 | 0.125 |
|  | *P_MCMC_*(intercept) | 0.109 | 0.058 | 0.340 |
|  | DIC | -182.82 | -184.75 | -186.25 |
| ES2 = log BM^0.33^ + log cochlea | log BM^0.33^ | 0.191 | -0.183 | 0.026 |
|  | log cochlea | -0.242 | 0.076 | 0.154 |
|  | intercept | 0.002 | 0.056 | -0.059 |
|  | *P_MCMC_*(log BM^0.33^) | <5 x 10^-4^ | 0.008 | 0.633 |
|  | *P_MCMC_*(log cochlea) | <5 x 10^-4^ | 0.374 | 0.070 |
|  | *P_MCMC_*(intercept) | 0.941 | 0.167 | 0.080 |
|  | DIC | -184.96 | -184.67 | -172.61 |
| ES3 = log BM^0.33^ | log BM^0.33^ | -0.042 | -0.196 | -0.107 |
|  | intercept | 0.016 | 0.086 | 0.047 |
|  | *P_MCMC_*(log BM^0.33^) | 0.295 | <5 x 10^-4^ | 0.033 |
|  | *P_MCMC_*(intercept) | 0.430 | 0.003 | 0.119 |
|  | DIC | -175.61 | -193.47 | -193.68 |
| ES3 = log BM^0.33^ + log cochlea | log BM^0.33^ | -0.036 | -0.222 | -0.098 |
|  | log cochlea | -0.021 | 0.082 | -0.024 |
|  | intercept | 0.021 | 0.069 | 0.051 |
|  | *P_MCMC_*(log BM^0.33^) | 0.402 | <5 x 10^-4^ | 0.094 |
|  | *P_MCMC_*(log cochlea) | 0.744 | 0.251 | 0.790 |
|  | *P_MCMC_*(intercept) | 0.414 | 0.024 | 0.131 |
|  | DIC | -174.05 | -188.31 | -189.17 |

Abbreviations: ES – eigenshape; BM – body mass; DIC – Deviance information criterion; SE – standard error; DF – degrees of freedom; *P* – probability; adj – adjusted.

**Table S5**

**A (i) Model comparison of the relationship between semicircular canal size and wing morphology (wing loading and wing aspect ratio) across all bat species sampled, taking into account the phylogenetic relatedness of species**

| Model tested | Parameters | ASCC | LSCC | PSCC |
| --- | --- | --- | --- | --- |
| log *R* = log BM^0.33^ | log BM^0.33^ | 0.492 | 0.477 | 0.488 |
|  | intercept | -0.277 | -0.320 | -0.337 |
|  | *P_MCMC_*(log BM^0.33^) | <1 x 10^-4^ | <1 x 10^-4^ | <1 x 10^-4^ |
|  | *P_MCMC_*(intercept) | <1 x 10^-4^ | <1 x 10^-4^ | <1 x 10^-4^ |
|  | DIC | -129.32 | -125.27 | -129.27 |
| log *R* = log BM^0.33^ + log WL | log BM^0.33^ | 0.459 | 0.414 | 0.433 |
|  | log WL | 0.030 | 0.056 | 0.051 |
|  | intercept | -0.295 | -0.352 | -0.367 |
|  | *P_MCMC_*(log BM^0.33^) | <1 x 10^-4^ | <5 x 10^-4^ | <1x 10^-4^ |
|  | *P_MCMC_*(log WL) | 0.664 | 0.455 | 0.464 |
|  | *P_MCMC_*(intercept) | <1 x 10^-4^ | <1 x 10^-4^ | <1 x 10^-4^ |
|  | DIC | -127.63 | -123.92 | -127.78 |
| log *R* = log BM^0.33^ + log WL + log WAR | log BM^0.33^ | 0.463 | 0.418 | 0.440 |
|  | log WL | 0.022 | 0.047 | 0.036 |
|  | log WAR | 0.068 | 0.085 | 0.145 |
|  | intercept | -0.344 | -0.413 | -0.472 |
|  | *P_MCMC_*(log BM^0.33^) | <1 x 10^-4^ | <5 x 10^-4^ | <1x 10^-4^ |
|  | *P_MCMC_*(log WL) | 0.749 | 0.526 | 0.598 |
|  | *P_MCMC_*(log WAR) | 0.641 | 0.586 | 0.295 |
|  | *P_MCMC_*(intercept) | 0.004 | 0.001 | <5x 10^-4^ |
|  | DIC | -125.90 | -122.23 | -127.16 |

(ii) Model comparison of the relationship between semicircular canal size and wing tip shape index across all bat species sampled, taking into account the phylogenetic relatedness of species

| Model tested | Parameters | ASCC | LSCC | PSCC |
| --- | --- | --- | --- | --- |
| log *R* = log WTS | log WTS | -0.005 | 0.038 | 0.003 |
|  | intercept | -0.064 | -0.121 | -0.128 |
|  | *P_MCMC_*(log WTS) | 0.951 | 0.667 | 0.980 |
|  | *P_MCMC_*(intercept) | 0.077 | 0.002 | 0.001 |
|  | DIC | -84.82 | -81.55 | -85.15 |

**B (i) Model comparison of the relationship between semicircular canal size and wing morphology (wing loading and wing aspect ratio) across all Yangochiroptera species sampled, taking into account the phylogenetic relatedness of species**

| Model tested | Parameters | ASCC | LSCC | PSCC |
| --- | --- | --- | --- | --- |
| log *R* = log BM^0.33^ | log BM^0.33^ | 0.540 | 0.574 | 0.580 |
|  | intercept | -0.279 | -0.362 | -0.378 |
|  | *P_MCMC_*(log BM^0.33^) | <1 x 10^-4^ | <1 x 10^-4^ | <1 x 10^-4^ |
|  | *P_MCMC_*(intercept) | <1 x 10^-4^ | <1 x 10^-4^ | <1 x 10^-4^ |
|  | DIC | -97.98 | -99.48 | -108.54 |
| log *R* = log BM^0.33^ + log WL | log BM^0.33^ | 0.521 | 0.468 | 0.396 |
|  | log WL | 0.011 | 0.063 | 0.111 |
|  | intercept | -0.283 | -0.385 | -0.420 |
|  | *P_MCMC_*(log BM^0.33^) | 0.007 | 0.017 | 0.019 |
|  | *P_MCMC_*(log WL) | 0.902 | 0.516 | 0.221 |
|  | *P_MCMC_*(intercept) | <1 x 10^-4^ | <1 x 10^-4^ | <1 x 10^-4^ |
|  | DIC | -96.33 | -98.05 | -107.30 |
| log *R* = log BM^0.33^ + log WL + log WAR | log BM^0.33^ | 0.500 | 0.455 | 0.381 |
|  | log WL | 0.046 | 0.086 | 0.133 |
|  | log WAR | -0.178 | -0.114 | -0.115 |
|  | intercept | -0.163 | -0.308 | -0.341 |
|  | *P_MCMC_*(log BM^0.33^) | 0.007 | 0.018 | 0.032 |
|  | *P_MCMC_*(log WL) | 0.663 | 0.422 | 0.181 |
|  | *P_MCMC_*(log WAR) | 0.315 | 0.545 | 0.478 |
|  | *P_MCMC_*(intercept) | 0.230 | 0.034 | 0.011 |
|  | DIC | -97.14 | -98.17 | -107.45 |

**(ii) Model comparison of the relationship between semicircular canal size and wing tip shape index across all Yangochiroptera species sampled, taking into account the phylogenetic relatedness of species**.

| Model tested | Parameters | ASCC | LSCC | PSCC |
| --- | --- | --- | --- | --- |
| log *R* = log WTS | log WTS | -0.113 | -0.111 | -0.115 |
|  | intercept | -0.068 | -0.136 | -0.153 |
|  | *P_MCMC_*(log WTS) | 0.083 | 0.118 | 0.088 |
|  | *P_MCMC_*(intercept) | 0.006 | <1 x 10^-4^ | <1 x 10^-4^ |
|  | DIC | -70.84 | -68.44 | -69.28 |

**C (i) Model comparison of the relationship between semicircular canal size and wing morphology (wing loading and wing aspect ratio) across all echolocating Yinpterochiroptera species sampled, taking into account the phylogenetic relatedness of species**

| Model tested | Parameters | ASCC | LSCC | PSCC |
| --- | --- | --- | --- | --- |
| log *R* = log BM^0.33^ | log BM^0.33^ | 0.361 | 0.358 | 0.355 |
|  | intercept | -0.256 | -0.267 | -0.264 |
|  | *P_MCMC_*(log BM^0.33^) | 0.105 | 0.161 | 0.159 |
|  | *P_MCMC_*(intercept) | 0.013 | 0.022 | 0.023 |
|  | DIC | -20.52 | -18.74 | -18.73 |
| log *R* = log BM^0.33^ + log WAR | log BM^0.33^ | 0.303 | 0.297 | 0.298 |
|  | log WAR | 0.296 | 0.288 | 0.277 |
|  | intercept | -0.472 | -0.475 | -0.466 |
|  | *P_MCMC_*(log BM^0.33^) | 0.192 | 0.262 | 0.256 |
|  | *P_MCMC_*(log WAR) | 0.364 | 0.441 | 0.451 |
|  | *P_MCMC_*(intercept) | 0.074 | 0.112 | 0.109 |
|  | DIC | -19.45 | -17.28 | -17.26 |
| log *R* = log BM^0.33^ + log WL + log WAR | log BM^0.33^ | 0.289 | 0.208 | 0.205 |
|  | log WL | 0.009 | 0.109 | 0.104 |
|  | log WAR | 0.305 | 0.337 | 0.335 |
|  | intercept | -0.483 | -0.588 | -0.581 |
|  | *P_MCMC_*(log BM^0.33^) | 0.336 | 0.538 | 0.539 |
|  | *P_MCMC_*(log WL) | 0.967 | 0.658 | 0.662 |
|  | *P_MCMC_*(log WAR) | 0.398 | 0.408 | 0.398 |
|  | *P_MCMC_*(intercept) | 0.191 | 0.159 | 0.153 |
|  | DIC | -17.59 | -15.73 | -15.75 |

**(ii) Model comparison of the relationship between semicircular canal size and wing tip shape index across all echolocating Yinpterochiroptera species sampled, taking into account the phylogenetic relatedness of species and including/excluding the potential outlier *Rhinolophus philippinensis*** small morph.

| Model tested | Parameters | ASCC | LSCC | PSCC |
| --- | --- | --- | --- | --- |
| log *R* = log WTS | log WTS | 0.264 | 0.336 | 0.264 |
|  | intercept | -0.193 | -0.224 | -0.230 |
|  | *P_MCMC_*(log WTS) | 0.110 | 0.066 | 0.120 |
|  | *P_MCMC_*(intercept) | 0.006 | 0.006 | 0.001 |
|  | DIC | -19.84 | -18.53 | -19.43 |
| log *R* = log WTS | log WTS | 0.350 | 0.401 | 0.345 |
| (excl. *R. philippinensis* small) | intercept | -0.205 | -0.233 | -0.240 |
|  | *P_MCMC_*(log WTS) | 0.024 | 0.009 | 0.025 |
|  | *P_MCMC_*(intercept) | 0.002 | 0.002 | 0.001 |
|  | DIC | -27.33 | -28.79 | -24.90 |

Abbreviations: *R* – Semicircular canal radius of curvature; BM – body mass; WL – wing loading; WAR – wing aspect ration; WTS – wing tip shape; *P_MCMC_* – probability; DIC – Deviance Information Criterion.

**Supplementary References:**

1. Welker KL, Orkin JD, Ryan TM (2009) Analysis of intraindividual and intraspecific variation in semicircular canal dimensions using high-resolution x-ray computed tomography. J Anat 215: 444–451.

2. Miller JD (2007) Sex differences in the length of the organ of Corti in humans. J Acoust Soc Am 121: EL151–EL155.

3. Kingston T, Rossiter SJ (2004) Harmonic-hopping in Wallacea's bats. Nature 429: 654–657.

4. Wiley DF (2007). Landmark Institute for Data Analysis and Visualization University of California, Davis. <http://www.idav.ucdavis.edu/research/EvoMorph>. Accessed 10 October 2008.

5. Spoor F, Bajpal S, Hussaim ST, Kumar K, Thewissen JGM (2002) Vestibular evidence for the evolution of aquatic behaviour in early cetaceans. Nature 417: 163–166.

6. Spoor F, Garland T, Krovitz G, Ryan TM, Silcox MT, et al. (2007) The primate semicircular canal system and locomotion. Proc Natl Acad Sci U S A 104: 10808–10812.

7. Cox PG, Jeffery N (2010) Semicircular canals and agility: the influence of size and shape measures. J Anat 216: 37–47.

8. R Development Core Team (2010). R: A language and environment for statistical computing. R Foundation for Statistical Computing Vienna, Austria. ISBN 3-900051-07-0 <http://www.R-project.org/>. Accessed 26 July 2010.

9. Knell RJ (2009) On the analysis of non-linear allometries. Ecol Entomol 34: 1–11.

10. Jones G (1999) Scaling of echolocation call parameters in bats. J Exp Biol 202: 3359–3367.

11. Maurer BA, Brown JH, Dayan T, Enquist BJ, Ernest SKM, et al. (2004) Similarities in body size distributions of small-bodied flying vertebrates. Evol Ecol Res 6: 783–797.

12. Dumont ER (2010) Bone density and the lightweight skeletons of birds. Proc R Soc B 277: 2193–2198.

13. Lohmann GP (1983) Eigenshape analysis of micro-fossils – a general morphometirc procedure for describing changes in shape. J Int Ass Math Geol 15: 659–672.

14. MacLeod N (1999) Generalizing and extending the eigenshape method of shape space visualization and analysis. Paleobiology 25: 107–138.

15. MacLeod N (2002) Geometric morphometrics and geological shape-classification systems. Earth-Sci Rev 59: 27–47.

16. Hammer Ø, Harper DAT, Ryan PD (2001) PAST: paleontological statistics software package for education and data analysis. Palaeontol Electron 4: 9.

17. Hadfield JD (2010) MCMC methods for multi-response generalized linear mixed models: The MCMCglmm R Package. J Stat Softw 33: 1–22.

18. Hadfield JD, Nakagawa S (2010) General quantitative genetic methods for comparative biology: phylogenies, taxonomies and multi-trait models for continuous and categorical characters. J Evol Biol 23: 494–508.

19. Wilson AJ, Reale D, Clements MN, Morrissey MM, Postma E, et al. (2009) An ecologist's guide to the animal model. J Anim Ecol 79: 13–26.

20. Norberg UM, Rayner JMV (1987) Ecological morphology and flight in bats (Mammalia; Chiroptera): wing adaptations, flight performance, foraging strategy and echolocation. Phil Trans R Soc B 316: 335–427.

21. Nummela S (1995) Scaling of the mammalian middle-ear. Hear Res 85: 18–30.

22. Norberg UM (1981) Allometry of bat wings and legs and comparison with bird wings. Phil Trans R Soc B 292: 359–398.

23. Zhang LB, Jones G, Zhang JS, Zhu GJ, Parsons S, et al. (2009) Recent surveys of bats (Mammalia: Chiroptera) from China. I. Rhinolophidae and Hipposideridae. Acta Chiropt 11: 71–88.

24. Sun KP, Feng J, Jiang TL, Ma J, Zhang ZZ, et al. (2008) A new cryptic species of *Rhinolophus macrotis* (Chiroptera: Rhinolophidae) from Jiangxi Province, China Acta Chiropt 10: 1–10.

25. Kingston T, Jones G, Zubaid A, Kunz TH (2000) Resource partitioning in rhinolophoid bats revisited. Oecologia 124: 332–342.

26. Olival KJ (2012) Evolutionary and ecological correlates of population genetic structure in bats. In: Gunnell GF, Simmons N, editors. Evolutionary History of Bats: Fossils, Molecules and Morphology. Cambridge: Cambridge University Press. pp. 267–316.

27. Struebig MJ (2005) Bat diversity and ecology in lowland forest, southern Borneo. Final report of the UEA Kalimantan Bat Expedition, 2004 1–19 p.

28. Smith AT, Xie Y, editors (2008) A guide to the mammals of China. Princeton: Princeton University Press. 576 p.

29. Aldridge HDJN, Rautenbach IL (1987) Morphology, echolocation and resource partitioning in insectivorous bats. J Anim Ecol 56: 763–778.

30. Barclay RMR, Brigham RM (1991) Prey detection, dietary niche breadth, and body size in bats - why are aerial insectivorous bats so small? Am Nat 137: 693–703.

31. Csada R (1996) *Cardioderma cor*. Mamm Species 519: 1–4.

32. Struebig MJ, Rossiter SJ, Bates PJJ, Kingston T, Oo SSL, et al. (2005) Results of a recent bat survey in Upper Myanmar including new records from the Kachin forests. Acta Chiropt 7: 147–163.

33. Borisenko A, Kruskop S (2003) Bats of Vietnam and adjacent territories. An identification manual: Geos. 202 p.

34. Smith F, Lyons S, Ernest S, Jones K, Kaufman D, et al. (2003) Body mass of late Quaternary mammals. Ecology 84: 3403.

35. Thabah A, Li G, Wang YN, Liang B, Hu KL, et al. (2007) Diet, echolocation calls, and phylogenetic affinities of the great evening bat (*Ia io;* Vespertilionidae): Another carnivorous bat. J Mammal 88: 728–735.

36. Thollesson M, Norberg UM (1991) Moments of inertia of bat wings and body. J Exp Biol 158: 19–35.

37. Mancina CA (2005) *Pteronotus macleayii*. Mamm Species 778: 1–3.

38. Rydell J, Arita HT, Santos M, Granados J (2002) Acoustic identification of insectivorous bats (order Chiroptera) of Yucatan, Mexico. J Zool 257: 27–36.

39. Mendellin RA, Arita HT (1989) *Tonatia evotis* and *Tonatia silvicola*. Mamm Species 334: 1–5.

40. Norberg UM, Fenton MB (1988) Carnivorous bats. Biol J Linn Soc 33: 383–394.

41. Yee D (2000) *Peropteryx macrotis*. Mamm Species 643: 1–4.
